# Efficient Multistate Free-Energy Calculations with QM/MM Accuracy Using Replica-Exchange Enveloping Distribution Sampling

**DOI:** 10.1021/acs.jpcb.5c02086

**Published:** 2025-06-06

**Authors:** Domen Pregeljc, Ramon J. R. Hügli, Sereina Riniker

**Affiliations:** Department of Chemistry and Applied Biosciences, ETH Zürich, Vladimir-Prelog-Weg 2, 8093 Zürich, Switzerland

## Abstract

Calculating free-energy differences using molecular dynamics (MD) simulations is an important task in computational chemistry. In practice, the accuracy of the results is limited by model approximations and insufficient phase-space sampling due to limited computational resources. In the present work, we address these challenges by integrating the quantum-mechanical/molecular-mechanical (QM/MM) scheme with replica-exchange enveloping distribution sampling (RE-EDS) to obtain a multistate and multiscale free-energy method with high computational efficiency. The performance of QM/MM RE-EDS is showcased by calculating hydration free energies for three data sets using semiempirical methods for the QM zone. We highlight the importance of the choice of QM Hamiltonian and the effect of the compatibility between the QM and MM models. Especially the choice of semiempirical method has a substantial effect on the accuracy compared to experiment, but also the choice of MM water model is non-negligible. Our findings indicate that RE-EDS is an efficient approach for calculating free-energy differences with a QM/MM scheme, and lays the foundation for future developments and applications.

## Graphical abstract

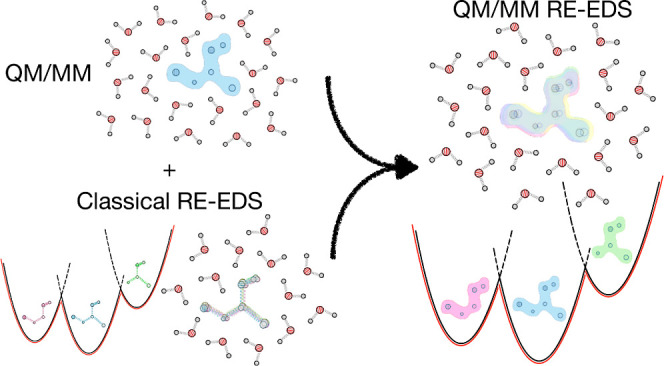

## Introduction

Accurate and reliable estimation of free-energy differences is one of the central objectives in computational chemistry with direct applications in various fields including drug discovery, physics, and materials science. For instance, a drug candidate should possess favorable physicochemical and pharmacokinetic properties as well as high potency and selectivity for its target.^1–3^ For the prediction of the latter properties, free-energy methods based on molecular dynamics (MD) simulations have been established as the state-of-the-art tool.^4–9^ Despite much progress in recent decades, some long-standing challenges remain unsolved.^10^ While MD simulations represent arguably the most rigorous way to estimate, for example, solvation and binding free energies, all-atom MD is associated with significant computational cost. Furthermore, the widely used classical fixed-charge force fields^11^ have known deficiencies.^12–15^ Therefore, a balance needs to be struck between the severity of approximations made in the model and the extent of phase-space sampling.^16,17^ Much ongoing research is focused on maximizing the achieved accuracy given the available computational resources by leveraging this trade-off.

While classical force fields have proven accurate enough in many applications (for examples see refs 18–20 ), there are cases where such approaches cannot reach sufficient accuracy. For instance, the fixed point-charge approximation employed in classical force fields does not hold for highly polarizable systems and/or environments. Quantum-mechanical (QM) methods would resolve this issue but even semiempirical approaches^21^ are typically too expensive to treat the full system for the simulation lengths needed in free-energy calculations (i.e., several nanoseconds). To reduce the computational costs while retaining the higher level of theory for the region of interest, a multiscale approach like QM/MM^22,23^ can be employed. Thereby, the studied system is divided into a QM zone, for which a QM description is used, while the surrounding environment is treated classically (MM zone).^24,25^ Multiscale approaches aim to combine the best of both worlds, see refs 26–36 for successful QM/MM applications.

However, even QM/MM when using density functional theory (DFT^37^) (or more accurate methods) is still (too) expensive for free-energy calculations. Thus, the common approach for QM/MM free-energy calculations, which goes back to Gao^26^ and Warshel,^38^ is to use a thermodynamic cycle with sampling at a lower level of theory (classical or using a semiempirical method) and employing free-energy perturbation (FEP)^39^ to estimate the higher-level QM/MM free-energy differences.^27,28,40^ The accuracy of this dual-resolution approach depends crucially on the extent of similarity between the potential-energy surfaces at the low and high levels of theory. If the surfaces are significantly different, the reweighting procedure fails to deliver converged values as the relevant regions of phase space for the high-level Hamiltonian are not sufficiently sampled.^41^ In the past decades, the main effort in QM/MM free-energy method development has been focused on this issue of insufficient overlap, and different strategies have been proposed to address it.^30,40,42–49^ Another alternative is to substitute the expensive QM calculations with a machine-learned interatomic potential (MLIP).^50–56^

Independent of the strategy used to improve the overlap in the dual-resolution framework, the free-energy methods commonly used are pairwise, i.e., a separate calculation for each pair of ligands needs to be carried out. A more efficient approach would be to use a multistate free-energy method, where multiple ligands are treated in a single simulation.

In this study, we combine the QM/MM scheme with the multistate method replica-exchange enveloping distribution sampling (RE-EDS)^57–60^ to perform free-energy calculations. The computational efficiency of (RE-)­EDS in a classical setting comes largely from the fact that the interactions between unperturbed particles in the system (i.e., the environment) have to be calculated only once—independent of the number of ligands (if a pairwise decomposable method like reaction field^61^ is used for the long-range electrostatic interactions).^60^ This reduction is less relevant in a QM/MM setting as the costs of the QM calculations dominate, but it becomes more important again if the expensive QM Hamiltonian is replaced by a cheaper MLIP^50–56^ in future applications. Furthermore, as with all multistate methods, there is no need for designing a perturbation map with a subset of the possible pairwise transformations. Compared to classical RE-EDS, the introduction of QM/MM allows for more accurate modeling of systems where polarization is important or which are not parametrized well by force fields. Related methods merging QM/MM schemes with enhanced sampling exist,^62,63^ however, this work is the first time RE-EDS is utilized with a multiscale approach and a framework for inclusion of any Hamiltonian for the perturbed part of the system is presented.

The developed QM/MM RE-EDS methodology is tested by calculating hydration free-energies for three different sets of molecules, for which experimental data is available,^64^ and the results are compared to those obtained using classical force fields. In addition, we investigate the effect of the choice of the semiempirical method and the MM water model on the agreement with experiment. Note that we employ semiempirical methods for the QM zone for speed reasons, although this limits the possible gain in accuracy over classical force fields.^65^ Nevertheless, the developed method is QM model agnostic and more accurate alternatives can be used. The results could of course be further processed in a dual-resolution framework, although this is not the focus here. Rather, we aim to describe the main theoretical and implementation aspects of QM/MM RE-EDS and demonstrate its practical applications.

The present work is structured as follows: in the Theory section a short introduction to QM/MM, and RE-EDS is given, followed by a detailed description of how the two methods were integrated into QM/MM RE-EDS. The developed methodology has been tested on three different systems, which are described in the Methods section, and the performance is evaluated in Results and Discussion. Finally, main findings and prospective work are summarized in the Conclusions.

## Theory

### QM/MM

Partitioning a system into a QM zone and an MM zone introduces a boundary between the zones. Here, we focus on the most relevant aspects and refer interested readers to excellent introductory texts for detailed descriptions.^24,25^ The potential energy of the entire system is typically constructed through the so-called additive scheme^24^

1 $$V^{QM/MM}\left(r^{QM},r^{MM}\right)=V^{QM}\left(r^{QM}\right)+V^{MM}\left(r^{MM}\right)+V^{QM-MM}\left(r^{QM},r^{MM}\right)$$

where **r** ^QM^ are the coordinates of the QM particles and **r** ^MM^ the coordinates of the MM particles. Within the QM/MM formalism, the interactions between the zones (*V* ^QM–MM^) can be treated at varying levels of complexity.^66,67^ Electrostatic embedding is employed in this work as it provides a superior trade-off between accuracy and computational cost in comparison to the simpler mechanical embedding and the more complex polarizable embedding schemes. In electrostatic embedding, MM particles within a predefined cutoff radius *R*_QM–MM_ enter the QM Hamiltonian as one-electron operators and can thus polarize the QM zone. van der Waals interactions between the QM and MM particles are calculated classically.

### Replica-Exchange Enveloping Distribution Sampling (RE-EDS)

Most widely used alchemical free-energy methods such as thermodynamic integration (TI)^68^ and free-energy perturbation (FEP)^39^ are pathway dependent. For a fully connected graph consisting of *N* end-states, *N*(*N* − 1)/2 separate simulations would need to be carried out in theory. Such an approach would, however, be redundant and there are estimates that a perturbation map with O(Nlog⁡N) scaling is sufficient.^69^ Even so, multistate methods offer a better computational efficiency as the interactions between unperturbed particles need to be calculated only once, independent of *N* (if the potential energy can be decomposed pairwise, e.g., using a reaction-field^61^ approach for the long-range electrostatic interactions).

Enveloping distribution sampling (EDS)^70–73^ is a multistate method, which uses a reference state, *V*_*R*_, that encompasses all end-states of interest and requires no definition of pathways between end-states. The reference state *V*_*R*_ is constructed in the following way

2 $$V_{R}\left(r;s,E^{R}\right)=-\frac{1}{\beta s}\ln\left[\sum_{i=1}^{N}e^{-\beta s\left(V_{i}\left(r\right)-E_{i}^{R}\right)}\right]$$

where $\beta ={\left(k_{B}T\right)}^{-1}$ with *k*_B_ being the Boltzmann constant and *T* the absolute temperature, and *V*_*i*_(**r**) is the potential energy of end-state *i*. The smoothness parameter *s* and the energy offsets **E** ^*R*^ are used to tune the reference state and achieve adequate sampling of all end-states. A smoothness parameter *s* = 1.0 results in a reference state that represents the “physically correct” description of the system. Reducing *s* (i.e., *s* < 1.0) smooths the energy barriers between end-states, thus facilitating transitions between them. At very small *s*-values, the potential-energy landscape is significantly disturbed and unphysical configurations are sampled (termed “undersampling”).^74^ Energy offsets **E** ^*R*^ are used to give equal weights to all end-states in the reference state.

In EDS, the force acting on a particle *k* is calculated as follows:

3 fk(t)=−∂VR(r;s,ER)∂rk=∑i=1Ne−βs(Vi(r)−EiR)∑j=1Ne−βs(Vj(r)−EjR)(−∂Vi(r)∂rk)=∑i=1Nwi(−∂Vi(r)∂rk)

The prefactor *w*_*i*_ determines the contributions of each end-state to the force acting on a particle. The larger the *s*-value, the more dominant is the end-state with the most favorable potential energy compared to all other end-states. Conversely, at very small *s*-values, all end-states contribute significantly to the forces.

From the simulation of the EDS reference state, all *N*(*N* − 1)/2 free-energy differences can be obtained through reweighting^70–73^ (i.e., using the Zwanzig equation^39^). For two end-states *A* and *B*, Δ*G*_*BA*_ is calculated as

4 ΔGBA=ΔGBR+ΔGRA=−1β(ln⁡⟨e−β(VB−VR)⟩R−ln⁡⟨e−β(VA−VR)⟩R)=−1βln⁡⟨e−β(VB−VR)⟩R⟨e−β(VA−VR)⟩R

Obtaining an optimal set of EDS reference-state parameters for more than two end-states proved to be a difficult task due to their mutual dependence.^74^ This issue can be partially circumvented by combining EDS with the replica-exchange technique to form replica-exchange enveloping distribution sampling (RE-EDS).^57–60,75–78^ RE-EDS is a form of Hamiltonian replica exchange, wherein replicas differ in the smoothness parameter *s*. Transitions between end-states are easier in replicas with small *s*-values, while physically relevant configurations are sampled in the top replica with *s* = 1.0. Exchanges between neighboring replicas *k* and *l* are accepted or rejected based on the Metropolis–Hastings criterion,^57^

5 $$p_{k,l}=\min\left(1,e^{-\beta \left(\left(H_{R}\left(r_{k};s_{l}\right)+H_{R}\left(r_{l};s_{k}\right)\right)-\left(H_{R}\left(r_{k};s_{k}\right)+H_{R}\left(r_{l};s_{l}\right)\right)\right)}\right)$$

An automatic pipeline has been developed and refined to estimate the energy offsets and optimize the distribution of the replicas in *s*-space.^60,75^ The RE-EDS pipeline consists of three phases, namely parameter exploration, parameter optimization, and production. During parameter exploration, a relevant configuration is generated for each end-state, and a lower bound for the *s*-value as well as initial energy offsets **E** ^*R*^ are determined. Subsequently, the *s*-value distribution and **E** ^*R*^ are optimized iteratively to achieve adequate sampling of all end-states at *s* = 1.0. Finally, a production run is performed to obtain relative free-energy differences between all end-states from a single simulation. For more details see refs 60 and 75 .

### Integrating QM/MM with RE-EDS

#### Definition of the EDS Reference State

Classical RE-EDS simulations can be conducted with either a hybrid or a dual-topology approach.^60,79^ For the combination with QM/MM, we follow the dual-topology approach with each end-state consisting of an entire molecule as this allows us to design the EDS reference state independent of the QM Hamiltonian. Let us expand eq 1 as

6 VQM/MM(rQM,rMM)=VQM(rQM)+VMM(rMM)+VQM−MM,EE(rQM,rMM)+VQM−MM,vdW(rQM,rMM)

where *V* ^QM^(**r** ^QM^, **r** ^MM^) is the potential energy of the QM zone and *V* ^MM^(**r** ^MM^) is the potential energy of the MM zone. *V* ^QM–MM,EE^(**r** ^QM^, **r** ^MM^) is the coupling between the QM and MM zones using electrostatic embedding (EE), which is defined as

$$V^{QM-MM,EE}\left(r^{QM},r^{MM}\right)=-\underset{j}{\sum }Z_{j}\int \frac{\rho \left(r\right)}{\left|r-R_{j}\right|}dr+\underset{n}{\sum }\underset{j}{\sum }\frac{Z_{n}Z_{j}}{\left|R_{n}-R_{j}\right|}$$ 7

where ρ­(**r**) is the electron density in the QM subsystem, *n* ∈ QM and runs over all QM-treated nuclei, and *j* ∈ MM and runs over all point charges within the cutoff *R*_QM–MM_. *Z*_*n*_ is the nuclear charge of the QM atom and *Z*_*j*_ is the point charge of the MM particle *j*. *V* ^QM–MM,vdW^(**r** ^QM^, **r** ^MM^) is the van der Waals (vdW) interaction between the two zones, which is calculated classically with a Lennard-Jones (LJ) potential-energy function,

8 $$V^{QM-MM,vdW}\left(r^{QM},r^{MM}\right)=\sum_{i}\sum_{j}\left(\frac{C_{12,ij}}{r_{ij}^{12}}-\frac{C_{6,ij}}{r_{ij}^{6}}\right)$$

where *i* ∈ QM and *j* ∈ MM. *C*_12_ and *C*_6_ are parametrized according to the force field of choice. In principle, point charges and LJ parameters should be reparameterized at each step of a QM/MM simulation, but it is rarely done in practice due to the associated computational cost and instead default force-field parameters are commonly used.^25^ In most QM/MM set-ups, there is a mismatch between the list of point charges included in the electrostatically embedded QM calculation (i.e., union of MM particles in the cutoff spheres of all QM particles) and the list of MM particles interacting with a given QM particle in classical LJ interactions (i.e., spherical cutoff around a QM single particle). If the LJ cutoff is large enough, the neglected interactions should be negligible. Note that *V* ^QM^(**r** ^QM^) and *V* ^QM–MM,EE^(**r** ^QM^, **r** ^MM^) cannot be separated in practice for technical reasons. For an EDS end-state *i*, we will therefore use their combination, *V*_*i*_^QM,EE^(**r**), in the following.

When constructing the EDS reference state (eq 2), a decision needs to be made on which interactions to perturb. While it may seem straightforward to define an end-state as *V*_*i*_(**r**) = *V*_*i*_^QM,EE^(**r**) + *V*_*i*_^MM,vdW^(**r**), a technical subtlety in EDS prevents that. Namely, only the lowest-energy end-state for a configuration contributes significantly to the forces (eq 3) at high *s*-values. Therefore, intramolecular potential-energy terms should not be perturbed, because otherwise the particles of the other end-states may drift apart and through each other, preventing further transitions between end-states. To circumvent this problem, we introduce an additional vacuum QM calculation to evaluate the intramolecular potential in the absence of the MM environment.

We construct the end-state potential-energy function as

9 $$V_{i}\left(r\right)=V_{i}^{QM,EE}\left(r\right)-V_{i}^{QM,vac}\left(r\right)+V_{i}^{MM,vdW}\left(r\right)$$

where *V*_*i*_^QM,EE^(**r**) is the QM-calculated potential energy of the end-state embedded in MM point charges, *V*_*i*_^QM,vac^(**r**) is the QM-calculated potential energy of the end-state in vacuum, and *V*_*i*_^MM,vdW^(**r**) is the classical van der Waals interaction of the end-state with the MM zone.

#### Definition of the Forces

The QM/MM RE-EDS system is propagated in time as follows:

1. Forces on the particles in the MM zone are calculated and applied according to the force field

10 $$f_{k_{MM}}\left(t\right)=-\frac{\partial V^{MM}\left(r\right)}{\partial r_{k_{MM}}}$$

2. QM-calculated forces in vacuum are applied to the QM zone for all end-states

11 $$f_{k_{QM}}\left(t\right)=-\frac{\partial V^{QM,vac}\left(r\right)}{\partial r_{k_{QM}}}$$

3. Contribution to the forces of each EDS end-state is calculated according to eq 3 using the reference-state potential-energy function *V*_*R*_(**r**; *s*, **E** ^*R*^) (eq 2) constructed from end-states *V*_*i*_(**r**) according to eq 9. Forces are then added to any particle included in the QM-MM coupling

12 $$f_{k_{QM-MM}}\left(t\right)+=\sum_{i=1}^{N}w_{i}\left(-\frac{\partial V_{i}\left(r\right)}{\partial r_{k_{QM-MM}}}\right)$$

## Methods

### Test Systems

Three different test systems with experimentally known hydration free energies (taken from the FreeSolv database^64^) were selected to assess the performance of the developed methodology and compare to classical results (Figure 1). Set A consists of benzene derivatives, which have already been studied using classical RE-EDS.^76^ We do not expect any sampling issues for this set. Sets B and C were selected to study the difference between force fields and semiempirical methods for sulfides, alcohols, and ethers. In addition, molecules in these two data sets are more flexible than in set A.

**Figure 1:**
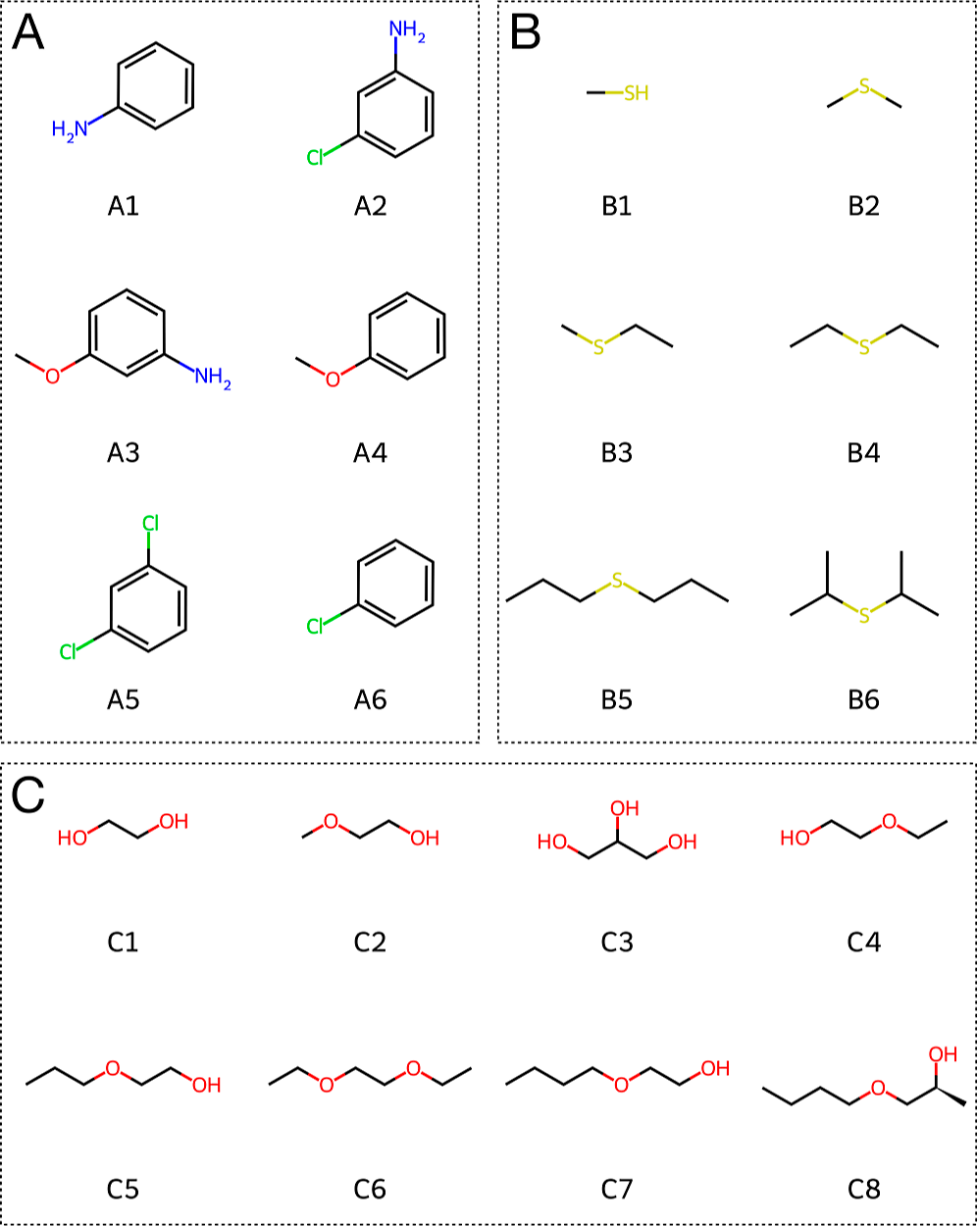
Data sets used to validate the developed QM/MM RE-EDS methodology. (A) Set A with benzene derivatives A1–A6. (B) Set B with sulfides B1–B6. (C) Set C with molecules C1–C8 containing hydroxy and ether groups.

### Simulation Details

All simulations were performed with a modified version of the GROMOS^80^ simulation package built upon an earlier GROMOS QM/MM implementation^81^ and the open-source Python3 ^82^ reeds^83^ module. QM/MM simulations were interfaced to xtb 6.5.1 ^84^ using GFN1-xTB^85^ and GFN2-xTB^86^ as well as to DFTB+ 24.1,^87^ using DFTB3 ^88^ with the hydrogen and the DFTD4 (using the D4 model^89,90^ ) dispersion corrections. Unless mentioned otherwise, default parameters were used. The water models TIP3P,^91^ SPC/E,^92^ and OPC3 ^93^ were used. A cutoff *R*_QM–MM_ = 1.2 nm was employed for the electrostatic embedding scheme. For the nonbonded interactions, a single 1.2 nm cutoff radius was used. Long-range nonbonded interactions were calculated using the reaction-field correction^61^ with ϵ_RF_ = 1 for vacuum simulations and ϵ_RF_ = 78.5 ^94^ for simulations in water. In the water simulations, a reference temperature of 298.15 K and pressure of 0.06102 kJ mol^–1^ nm^–3^ were maintained using the Berendsen thermostat and barostat.^95^ The stochastic dynamics integrator was used for the vacuum simulations. The SHAKE algorithm^96^ with a relative tolerance of 10^–4^ was used to constrain all bonds in the MM zone. The pairlist was updated every five steps. A time step of 0.5 and 2 fs was used for the QM/MM and MM simulations, respectively.

### Set A

Generalized AMBER force field (GAFF) 1.7 ^97^ topologies and coordinates were taken from ref 76. To ensure that the molecules remain well-aligned during the simulations, distance restraints with a force constant of 5000 kJ mol^–2^ nm^–2^ were applied on four atoms per molecule. For each end-state, a relevant end-state configuration was obtained by running a 1 ns long EDS simulations where **E** ^*R*^ was biased toward the given end-state by setting **E**_*i*_^*R*^ = 500 kJ mol^–1^ for end-state *i* and **E**_*j*_^*R*^ = −500 kJ mol^–1^ for all other end-states *j*. These optimized configurations were used in a starting-state mixing approach as described by Ries et al.^75^ The lower bound for *s* was determined using 21 parallel 0.2 ns EDS simulations with logarithmically distributed *s*-values between 1 and 10^–5^ and **E** ^*R*^ set to zero for all end-states. Next, initial energy offsets were estimated from a 0.5 ns RE-EDS simulation, followed by *s*-distribution optimization step to achieve round trips between the replicas and a subsequent energy-offset rebalancing step. With the optimal set of parameters, a 5 ns production run was performed (ten repeats with different random number seeds for the initial velocities).

The described procedure was carried out for classical RE-EDS simulations in water and in vacuum as well as for the newly developed QM/MM RE-EDS in water.

### Sets B and C

Molecules were parametrized with the OpenFF 2.0.0 force field.^98^ Topologies in the AMBER format were generated using the OpenFF Toolkit^99^ and subsequently converted to the GROMOS topology format using the GROMOS++^100^ program *amber2gromos*.^76^ GROMOS++ programs *pdb2g96* and *sim_box* were used to generate solvated boxes, and the programs *red_top* and *prep_eds* to prepare single molecule and perturbed topologies. To ensure that the molecules remain aligned during the simulation, distance restraints with a force constant of 5000 kJ mol^–2^ nm^–2^ were applied on two atoms per molecule (no restriction of any dihedral angle sampling). The same RE-EDS procedure as described for set A was carried out for these sets with only a small modification: separate *s*-optimization and energy-offset rebalancing steps were substituted by a single mixed optimization step as this was shown to be more effective.^60^ Ten iterations of 0.5 ns RE-EDS simulations were performed in which the *s*-value distribution and **E** ^*R*^ were optimized according to the N-GRTO algorithm.^58^ With the optimal set of parameters, a 5 ns production run was performed (ten repeats with different random number seeds for the initial velocities).

The described procedure was carried out for classical RE-EDS simulations in water and in vacuum as well as for the newly developed QM/MM RE-EDS in water.

### Analysis

All simulations were analyzed using GROMOS++ programs and PyGromosTools.^101^ For further analysis and visualization, the following Python packages were used: Matplotlib 3.7.1,^102^ mpmath 1.3.0,^103^ NumPy 1.24.3,^104^ Pandas 2.0.1,^105^ SciPy 1.10.1,^106^ and pymbar 4.0.3.^107^

Relative hydration free-energies between two end-states *i* and *j* were obtained based on a classical RE-EDS simulation in water and one in vacuum using the thermodynamic cycle

13 $$\Delta \Delta G_{ji}^{hyd}=\Delta G_{j}^{hyd}-\Delta G_{i}^{hyd}=\Delta G_{ji}^{wat}-\Delta G_{ji}^{vac}$$

Note that in QM/MM RE-EDS, we define the end-states to be exclusively characterized by their nonbonded interactions with the environment (see Theory section). Therefore, the relative free energy of two end-states in vacuum is by definition zero, i.e., Δ*G*_*ji*_^vac^ = 0. ΔΔ*G*_*ji*_^hyd^ can thus be obtained from a single QM/MM RE-EDS simulation in water

$$\Delta \Delta G_{ji}^{hyd}=\Delta G_{j}^{hyd}-\Delta G_{i}^{hyd}=\Delta G_{ji}^{\mathrm{wat}}$$ 14

Absolute hydration free energies (Δ*G* ^hyd^) were retrieved in the following way

$$\Delta G_{i}^{hyd}=\Delta \Delta G_{\mathrm{Ri}}^{hyd}-\left(\frac{\overset{N}{\underset{j}{\sum }}\Delta \Delta G_{\mathrm{Rj}}^{hyd}}{N}-\frac{\overset{N}{\underset{j}{\sum }}\Delta G_{j}^{\exp}}{N}\right)$$ 15

where *i* is the molecule of interest, *j* runs over all molecules, *R* is the reference state, and *N* is the number of molecules in a set.

Performance of the different methods was quantified using mean absolute error (MAE) and Kendall’s τ coefficient.^108^ Additional metrics, namely root-mean-square error (RMSE), Spearman’s ρ coefficient,^109^ the coefficient of determination (*R* ^2^) and Pearson correlation coefficient^110^ are given in the Supporting Information.

Section S5 in the Supporting Information provides RE-EDS-related plots, tracking the parameter optimization procedure, trajectory paths, sampling distribution and convergence of production runs (Figures S7–S16).

## Results and Discussion

We tested the developed QM/MM RE-EDS methodology by calculating hydration free energies of three data sets with small molecules (Figure 1). First, the QM/MM RE-EDS results are compared to computed results using classical RE-EDS and multistate Bennett acceptance ratio estimator (MBAR)^111,112^ as well as to experimental values (both taken from the FreeSolv database^64^). Subsequently, we showcase the effect of the choice of semiempirical method and water model on the QM/MM RE-EDS results.

### QM/MM RE-EDS Validation

For set A, we compared QM/MM RE-EDS (semiempirical method GFN2-xTB^86^ and the TIP3P^91^ water model) with the classical (MM) approaches RE-EDS and MBAR (both GAFF 1.7 ^97^) against the experimental values (Figure 2B and Table 1). As shown previously,^76^ RE-EDS gives equivalent results to other methods like MBAR when the same force field is used. The deviation from experiment is shown in Figure 2A. While all three approaches have MAE values below chemical accuracy and the two classical methods perform equivalently, QM/MM with GFN2-xTB yields a higher MAE than the classical force field. However, it provides a better ranking for this set of compounds.

**Figure 2:**
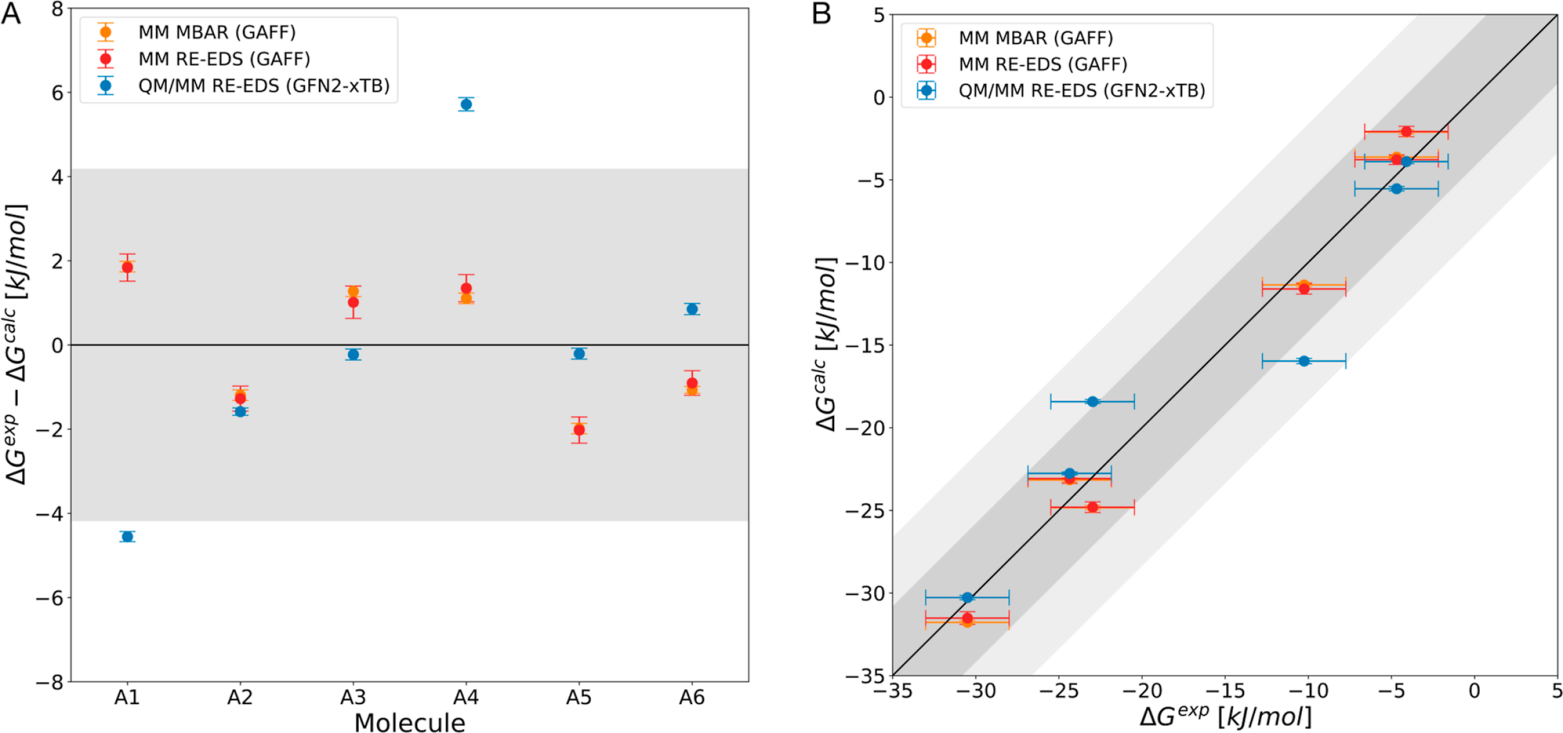
Results on set A for QM/MM RE-EDS (GFN2-xTB^86^ and TIP3P^91^), classical (MM) RE-EDS and MBAR (both GAFF 1.7 ^97^). (A) Comparison against experimental values taken from the FreeSolv database.^64^ (B) Correlation with experiment. Error bars represent the standard deviation over ten repeats and experimental uncertainty, respectively. Shaded gray area depicts the range that falls within ±4.184 kJ/mol (±1 kcal/mol) from perfect correlation and the dark gray area corresponds to an error margin of ±8.368 kJ/mol (±2 kcal/mol). Hydration free energies as a function of the molecule identifier are shown in Figure S1 in the Supporting Information.

**Table 1:** Mean Absolute Error (MAE) and Kendall’s τ for All Sets^a^

set	method	MAE [kJ/mol]	Kendall’s τ
A	MM MBAR (GAFF)	1.4	0.87
	MM RE-EDS (GAFF)	1.4	0.87
	QM/MM RE-EDS (GFN2-xTB)	2.2	1.00
B	MM MBAR (GAFF)	1.4	–0.60
	MM RE-EDS (OpenFF)	1.4	–0.60
	QM/MM RE-EDS (GFN2-xTB)	2.9	–0.33
C	MM MBAR (GAFF)	2.4	0.86
	MM RE-EDS (OpenFF)	2.3	0.79
	QM/MM RE-EDS (GFN2-xTB)	5.5	0.29

a Additional metrics are provided in Table S1 in the Supporting Information.

The sulfides in set B have very similar hydration free energies between −5 and −7 kJ/mol, thus challenging the accuracy limit of computational methods. As can be seen in Figure 3, the classical force fields GAFF and OpenFF give similar results and reproduce the experimental values well (within ±2 kJ/mol), except for compound B1 with a deviation of 3.2 kJ/mol. The error metrics are listed in Table 1. Due to the nearly identical hydration free energies of the compounds, the ranking (Kendall’s τ) does not contain much information. As for set A, all MAE values are below chemical accuracy and the QM/MM results with GFN2-xTB are worse than the classical force fields. There even appears to be systematic deviation as a function of the molecule size.

**Figure 3:**
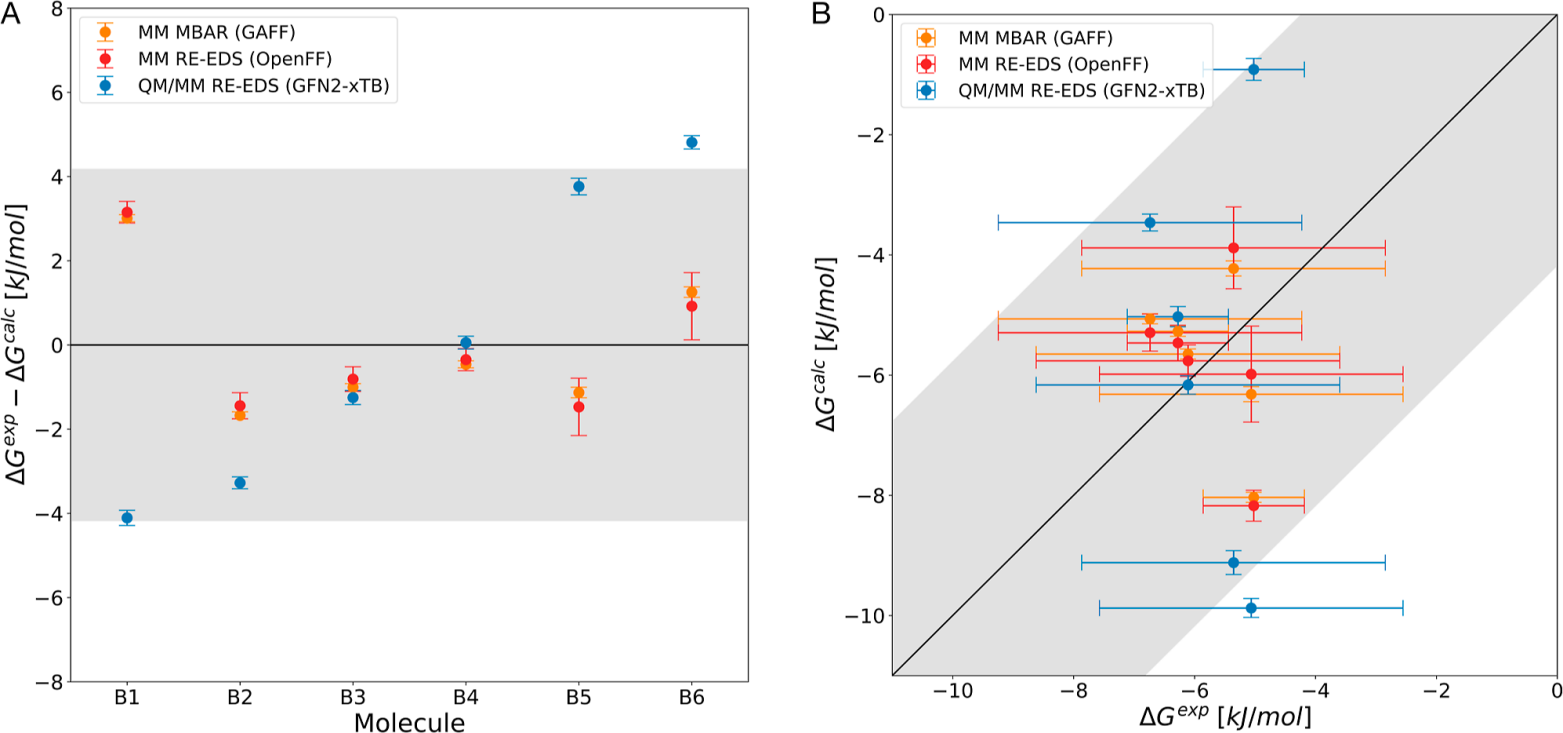
Results on set B for QM/MM RE-EDS (GFN2-xTB^86^ and TIP3P^91^), classical (MM) RE-EDS (OpenFF 2.0.0 ^98^) and MBAR (GAFF 1.7 ^97^). (A) Comparison against experimental values taken from the FreeSolv database.^64^ (B) Correlation with experiment. Error bars represent the standard deviation over ten repeats and experimental uncertainty, respectively. Shaded gray area depicts the range that falls within ±4.184 kJ/mol (±1 kcal/mol) from perfect correlation and the dark gray area corresponds to an error margin of ±8.368 kJ/mol (±2 kcal/mol). Hydration free energies as a function of the molecule identifier are shown in Figure S2 in the Supporting Information.

Similar to set B, the compounds in set C have a higher flexibility compared to set A, but in this case the hydration free energies span a much larger range of 50 kJ/mol. Figure 4 shows more variation between the force fields, although both give an MAE value below chemical accuracy and both again outperform QM/MM with GFN2-xTB (Table 1). In the case of set C, also the ranking as measured with Kendall’s τ is better with the classical force fields.

**Figure 4:**
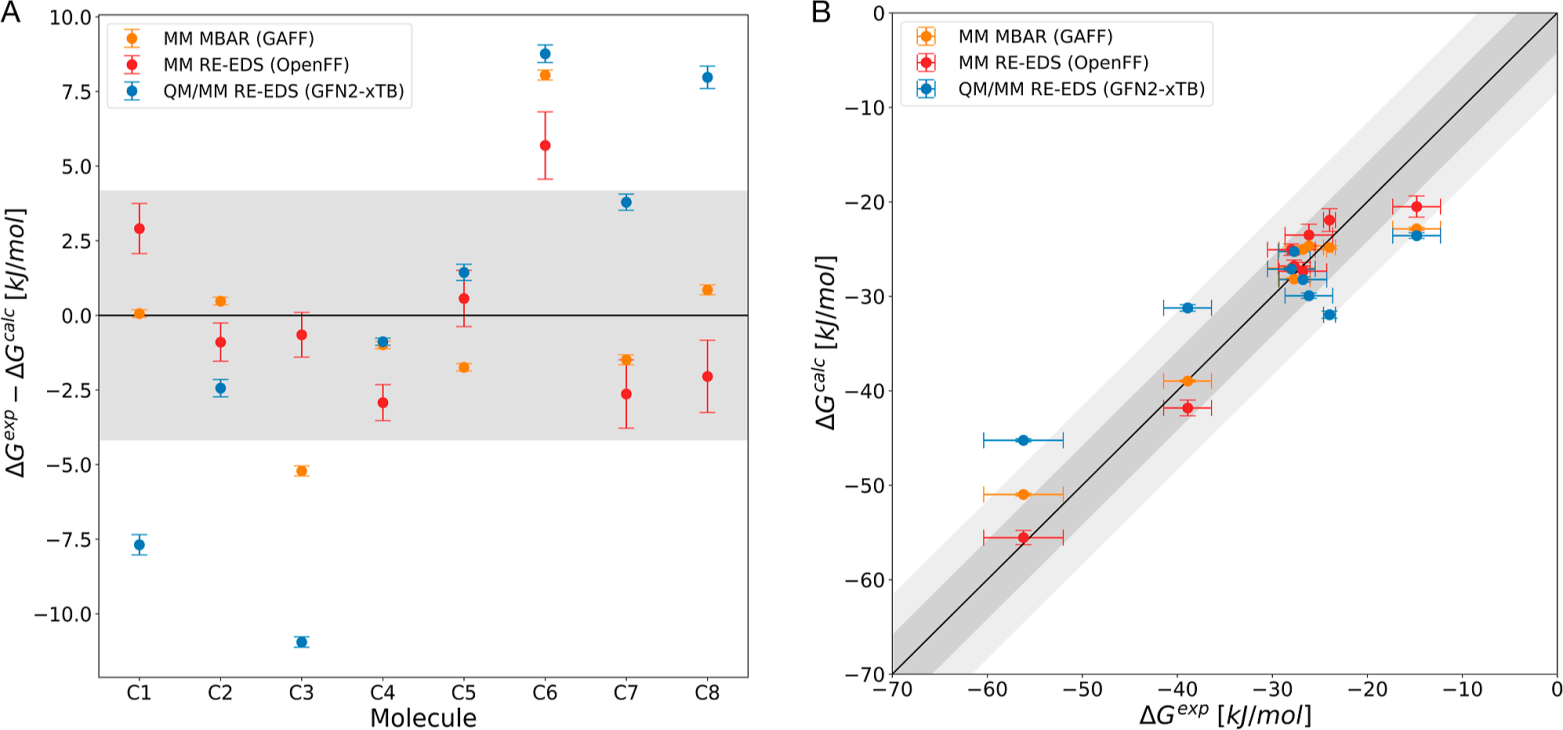
Results on set C for QM/MM RE-EDS (GFN2-xTB^86^ and TIP3P^91^), classical (MM) RE-EDS (OpenFF 2.0.0 ^98^) and MBAR (GAFF 1.7 ^97^). (A) Comparison against experimental values taken from the FreeSolv database.^64^ (B) Correlation with experiment. Error bars represent the standard deviation over ten repeats and experimental uncertainty, respectively. Shaded gray area depicts the range that falls within ±4.184 kJ/mol (±1 kcal/mol) from perfect correlation and the dark gray area corresponds to an error margin of ±8.368 kJ/mol (±2 kcal/mol). Hydration free energies as a function of the molecule identifier are shown in Figure S3 in the Supporting Information.

While the results show that RE-EDS can be used in a QM/MM setup to provide converged free-energy differences, we do not observe the expected improvement in accuracy when going from a fully classical description to QM/MM—in contrary. This may be due to the choice of QM Hamiltonian^65^ and the compatibility between the QM and MM models (see below). However, it is also worth pointing out that classical force fields are parametrized and/or validated using hydration free energies (as evidenced by the small deviations from experiment for the classical force fields).

### Choice of QM Hamiltonian

The choice of QM Hamiltonian can have a significant effect on the accuracy of results.^113^ To demonstrate this, we investigated the effect of the choice of QM Hamiltonian by performing QM/MM RE-EDS simulations with different semiempirical methods. Figure 5 shows the results for GFN1-xTB and GFN2-xTB for sets A and B. For set C, three methods GFN1-xTB, GFN2-xTB, and DFTB3 were compared and the results are provided in Figure 6. Table 2 lists the corresponding MAE and Kendall’s τ values.

**Figure 5:**
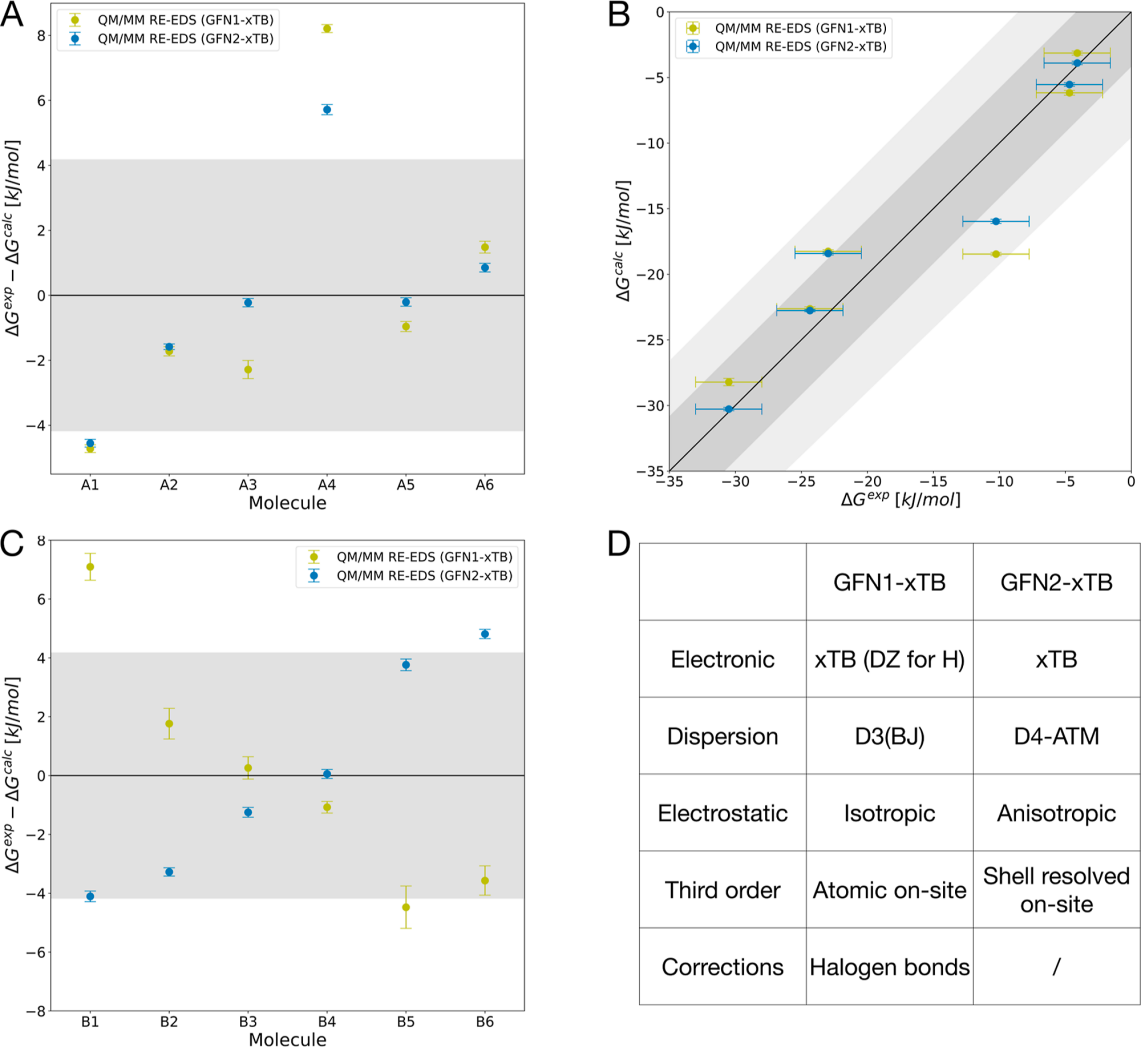
Comparison of QM/MM RE-EDS results with GFN1-xTB (yellow) and GFN2-xTB (blue) in TIP3P for sets A and B. (A) Deviation from experiment for set A. (B) Correlation to experiment for set A. (C) Deviation from experiment for set B. (D) Main differences between GFN1-xTB and GFN2-xTB methods. Error bars represent the standard deviation over repeats and experimental uncertainty for calculated values and experiment, respectively. Shaded light gray area depicts the range that falls within ±4.184 kJ/mol (±1 kcal/mol) from perfect correlation and the dark gray area corresponds to an error margin of ±8.368 kJ/mol (±2 kcal/mol). Hydration free energies as a function of the molecule identifier are shown in Figure S4 in the Supporting Information.

**Figure 6:**
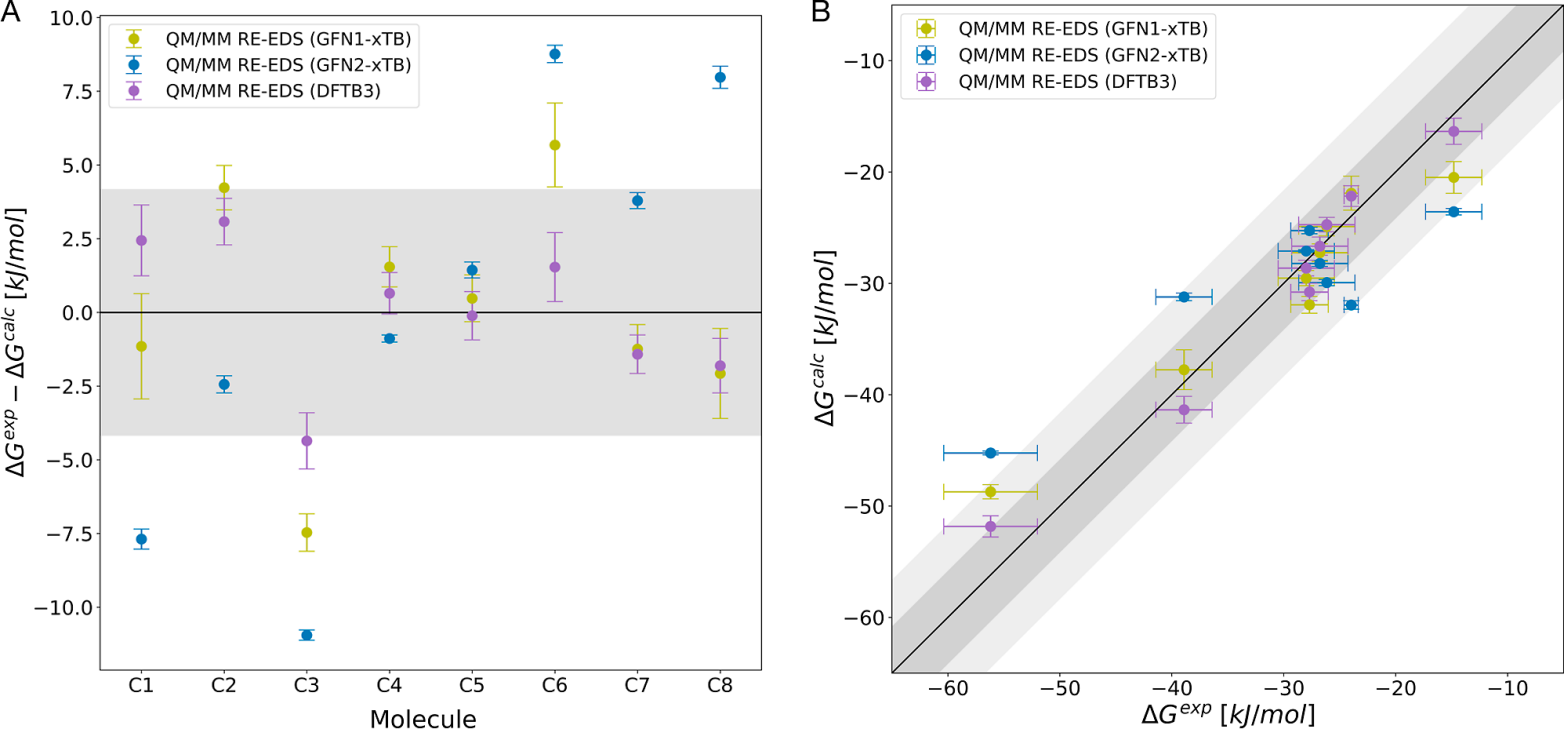
Comparison of QM/MM RE-EDS results with GFN1-xTB (yellow), GFN2-xTB (blue), and DFTB3 (purple) in TIP3P for set C. (A) Deviation from experiment for set C. (B) Correlation to experiment for set C. Error bars represent the standard deviation over repeats and experimental uncertainty for calculated values and experiment, respectively. Shaded light gray area depicts the range that falls within ±4.184 kJ/mol (±1 kcal/mol) from perfect correlation and the dark gray area corresponds to an error margin of ±8.368 kJ/mol (±2 kcal/mol). Hydration free energies as a function of the molecule identifier are shown in Figure S5 in the Supporting Information.

**Table 2:** Comparison of MAE and Kendall’s τ for All Sets When Varying the QM Hamiltonian in the QM/MM RE-EDS Simulations^a^

set	QM Hamiltonian	MAE [kJ/mol]	Kendall’s τ
A	GFN1-xTB	3.2	0.87
	GFN2-xTB	2.2	1.00
B	GFN1-xTB	3.0	0.20
	GFN2-xTB	2.9	0.33
C	GFN1-xTB	3.0	0.93
	GFN2-xTB	5.5	0.29
	DFTB3	1.9	0.93

a Additional metrics are provided in Table S2 in the Supporting Information.

From the results, it is evident that the choice of QM Hamiltonian (even when staying within the semiempirical methods) significantly affects the accuracy. In addition, the effect appears to be system dependent. For sets A and B, GFN1-xTB gives slightly worse results than GFN2-xTB, while for set C it clearly outperforms GFN2-xTB. In case of set B, the molecule size dependent deviation from experiment is opposite between GFN1-xTB and GFN2-xTB, suggesting a parametrization-related source of error. For set C, DFTB3 shows best agreement with experiment, well within chemical accuracy, while the relative ranking is the same as for GFN1-xTB.

### Compatibility of QM and MM Models

In addition to the choice of QM Hamiltonian, also the compatibility of models used in QM/MM simulations effects the accuracy of the results.^114–117^ To assess this influence, we studied the effect of the choice of MM water model for set C, comparing the compatibility of TIP3P,^91^ SPC/E,^92^ and OPC3 ^93^ with GFN1-xTB and GFN2-xTB. Parameters and selected properties of these models are shown in Table S3 in the Supporting Information.

As can be seen in Figure 7 and Table 3, the effect of the choice of MM water model is not as significant as the difference between the QM Hamiltonians, but it affects the results nevertheless. For both GFN-xTB variants, the SPC/E water model appears to be most compatible, yielding the lowest MAE values.

**Figure 7:**
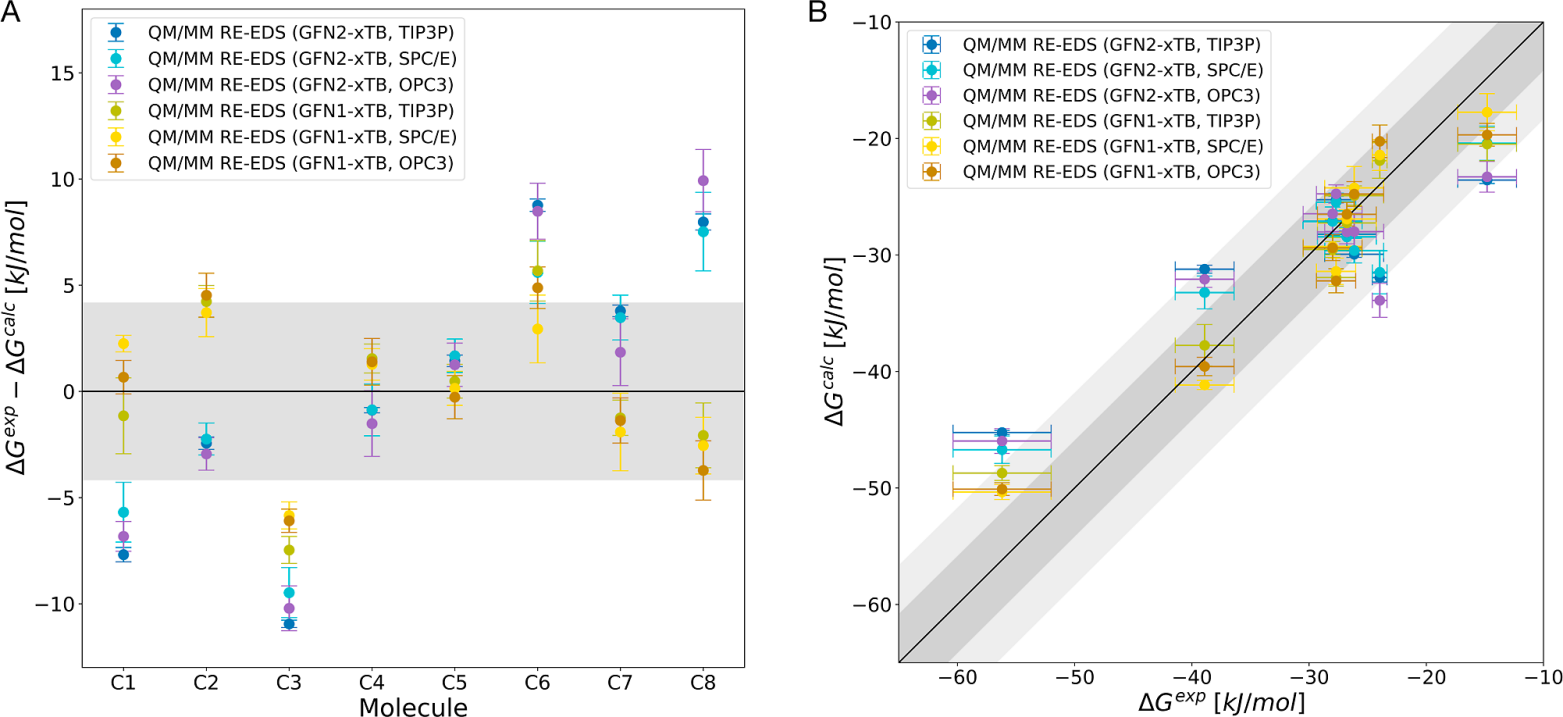
Comparison of QM/MM RE-EDS results with GFN1-xTB (yellow lines) and GFN2-xTB (blue lines) in TIP3P, SPC/E, and OPC3 (shades of colors) for set C. (A) Deviation from experiment for set C. (B) Correlation to experiment for set C. Error bars represent the standard deviation over repeats and experimental uncertainty for calculated values and experiment, respectively. Shaded light gray area depicts the range that falls within ±4.184 kJ/mol (±1 kcal/mol) from perfect correlation and the dark gray area corresponds to an error margin of ±8.368 kJ/mol (±2 kcal/mol). Hydration free energies as a function of the molecule identifier are shown in Figure S6 in the Supporting Information.

**Table 3:** Comparison of MAE and Kendall’s τ for Set C When Varying MM Water Model and the QM Hamiltonian in the QM/MM RE-EDS Simulations^a^

QM Hamiltonian	water model	MAE [kJ/mol]	Kendall’s τ
GFN1-xTB	TIP3P	3.0	0.93
	SPC/E	2.6	0.93
	OPC3	2.9	0.93
GFN2-xTB	TIP3P	5.5	0.29
	SPC/E	4.6	0.36
	OPC3	5.4	0.36

a Additional metrics are provided in Table S3 in the Supporting Information.

## Conclusions

In this work, we introduced the combination of the QM/MM scheme with the multistate free-energy method RE-EDS. By doing this, we may increase the accuracy for highly polarizable systems and extend the applicability domain of RE-EDS to systems not parametrized by classical force fields. As a proof-of-concept, we tested the performance of QM/MM RE-EDS by calculating hydration free energies for three data sets using semiempirical methods for the QM Hamiltonian. For all systems, we obtained converged results, which contained up to eight end-states simultaneously. Classical force fields outperformed the QM/MM treatment for two out of three data sets, which has been seen before and is not necessarily surprising because hydration free energies are used within the parametrization pipelines of most force fields. The performance of QM/MM RE-EDS depended strongly on the choice of semiempirical method, with DFTB3 giving the best results of all tested methods (including classical force fields) for set C. Furthermore, we investigated the importance of the compatibility of the QM and MM models in the QM/MM scheme by comparing three different MM water models. The variability is smaller in this case, albeit still non-negligible with up to 3.4 kJ/mol.

While we used semiempirical methods for the QM zone for speed reasons, there is no restriction on the QM Hamiltonian used in QM/MM RE-EDS. Possible future directions include further optimization of the compatibility between the QM and MM models, substituting the QM model by a MLIP for ML/MM calculations, and applying the methodology to study problems with a larger polarization effect.

## Supplementary Material



## Data Availability

The developed QM/MM RE-EDS methodology has been implemented in the GROMOS software package and the source code is available on GitHub: https://github.com/rinikerlab/gromosXX/tree/qmmm_reeds. All the input files required to reproduce this work are available on GitHub: https://github.com/rinikerlab/qmmm_reeds.

## Notes

Extended set of error metrics, and additional figures and tables referenced in the text (PDF)
