# Supplementary material for: Efficient Multistate Free-Energy Calculations with QM/MM Accuracy Using Replica-Exchange Enveloping Distribution Sampling
Source: J Phys Chem B. 2025 Jun 6;129(24):5948–60. doi: 10.1021/acs.jpcb.5c02086 (PMC12183765; doi:10.1021/acs.jpcb.5c02086)
Supplement: Supplementary file 1 [file jp5c02086_si_001.pdf]

SUPPORTING INFORMATION
Efficient Multistate Free-Energy Calculations with QM/MM Accuracy using Replica-Exchange Enveloping Distribution Sampling

Domen Pregeljc[a], Ramon J. R. Hügli[a] and Sereina Riniker*[a]

[a] *Department of Chemistry and Applied Biosciences, ETH Zürich, Vladimir-Prelog-Weg 2, 8093 Zürich, Switzerland. E-mail: sriniker@ethz.ch*

Contents

S1 Validation of QM/MM RE-EDS **S2**

S2 Choice of QM Hamiltonian **S5**

S3 Compatibility of QM and MM Models **S7**

S4 Numerical Values **S8**

S5 RE-EDS Related Figures **S9**
S5.1 QM/MM RE-EDS with GFN2-xTB + TIP3P QM/MM for Set A S9
S5.2 QM/MM RE-EDS with GFN2-xTB + SPC/E for Set C S15

S1 Validation of QM/MM RE-EDS

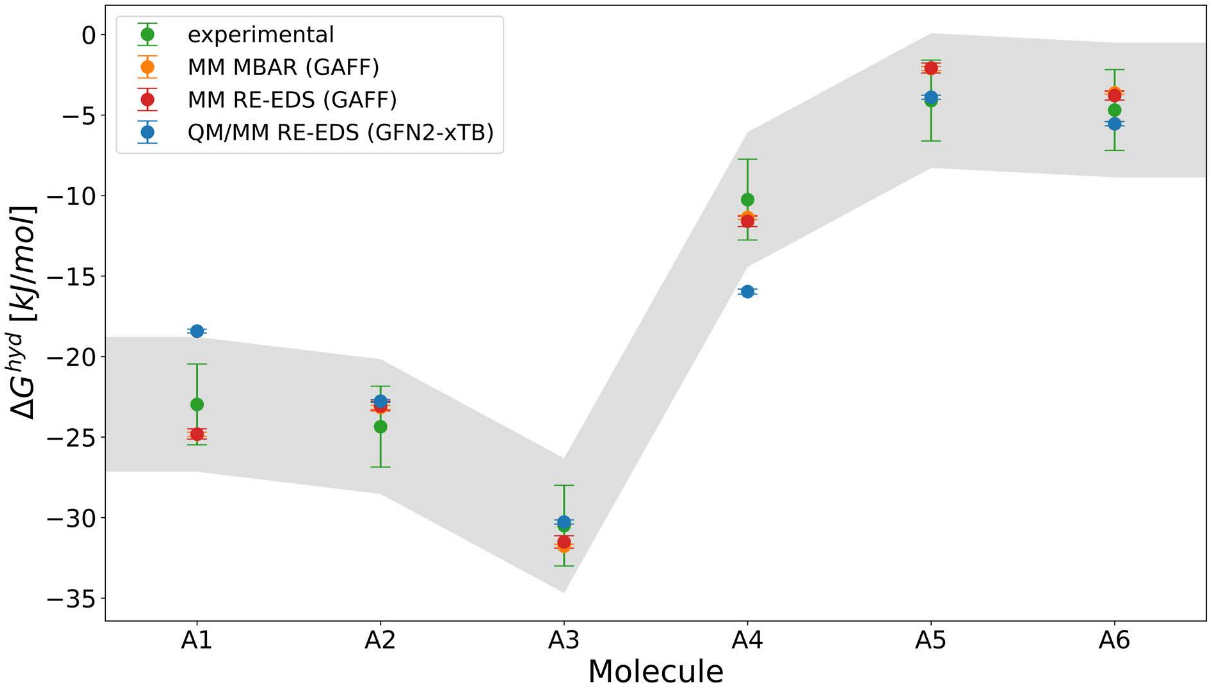

Figure S1: Hydration free energy as a function of the molecule identifier for set A for experiment (green), classical (MM) MBAR (orange) and RE-EDS (red) (both GAFF 1.7), and QM/MM RE-EDS (blue, GFN2-xTB and TIP3P). Error bars represent the standard deviation over repeats and experimental uncertainty for calculated values and experiment, respectively. The shaded grey area depicts the range that falls within ± 4.184 kJ/mol (± 1 kcal/mol) from experimental mean.

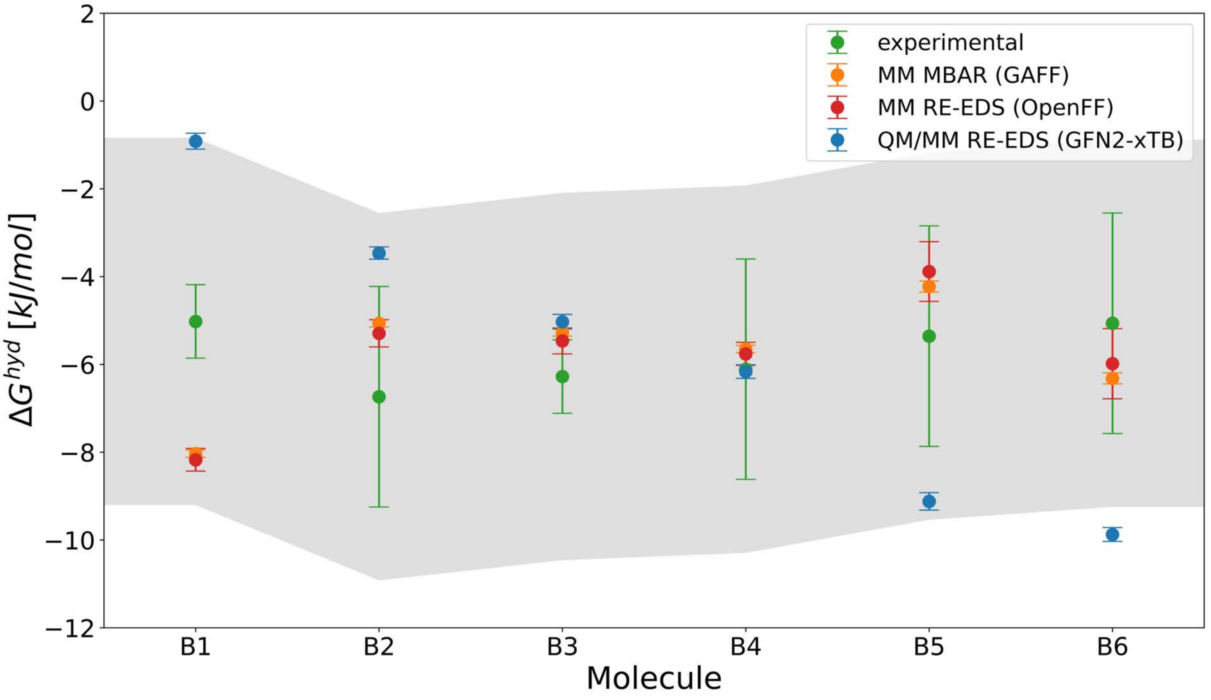

Figure S2: Hydration free energy as a function of the molecule identifier for set B for experiment (green), classical (MM) MBAR (orange, GAFF 1.7) and RE-EDS (red, OpenFF 2.0.0), and QM/MM RE-EDS (blue, GFN2-xTB and TIP3P). Error bars represent the standard deviation over repeats and experimental uncertainty for calculated values and experiment, respectively. Hydration free-energies. The shaded grey area depicts the range that falls within ± 4.184 kJ/mol (± 1 kcal/mol) from experimental mean.

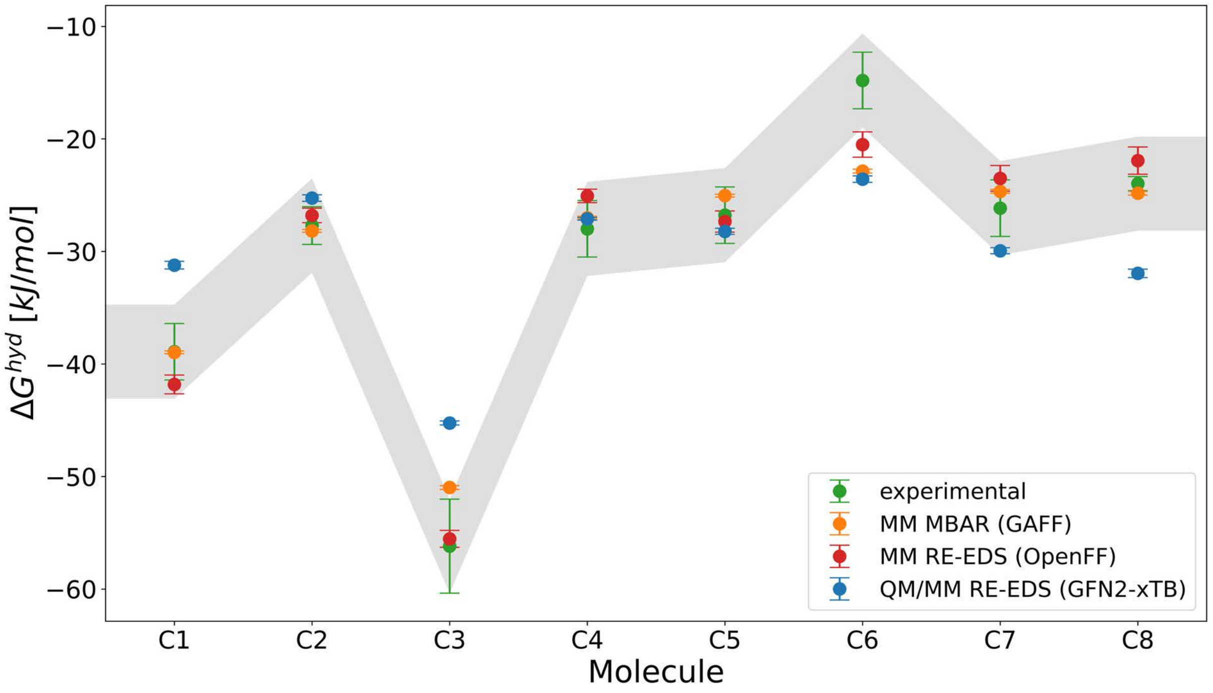

Figure S3: Hydration free energy as a function of the molecule identifier for set C for experiment (green), classical (MM) MBAR (orange, GAFF 1.7) and RE-EDS (red, OpenFF 2.0.0), and QM/MM RE-EDS (blue, GFN2-xTB and TIP3P). Error bars represent the standard deviation over repeats and experimental uncertainty for calculated values and experiment, respectively. The shaded grey area depicts the range that falls within $\pm$ 4.184 kJ/mol ($\pm$ 1 kcal/mol) from experimental mean.

Set	Method	RMSE [kJ/mol]	R^2	Spearman's ρ	Pearson correlation
A	MM MBAR (GAFF)	1.5	0.98	0.94	0.99
	MM RE-EDS (GAFF)	1.5	0.98	0.94	0.99
	QM/MM RE-EDS (GFN2-xTB)	3.1	0.91	1.0	0.96
B	MM MBAR (GAFF)	1.6	-5.29	-0.66	-0.52
	MM RE-EDS (OpenFF)	1.6	-5.23	-0.66	-0.36
	QM/MM RE-EDS (GFN2-xTB)	3.3	-25.19	-0.14	-0.26
C	MM MBAR (GAFF)	3.5	0.91	0.95	0.97
	MM RE-EDS (OpenFF)	2.8	0.94	0.90	0.97
	QM/MM RE-EDS (GFN2-xTB)	6.5	0.68	0.43	0.90

Table S1: Comparison of RMSE, R^2, Spearman's ρ and Pearson correlation for all sets.

S2 Choice of QM Hamiltonian

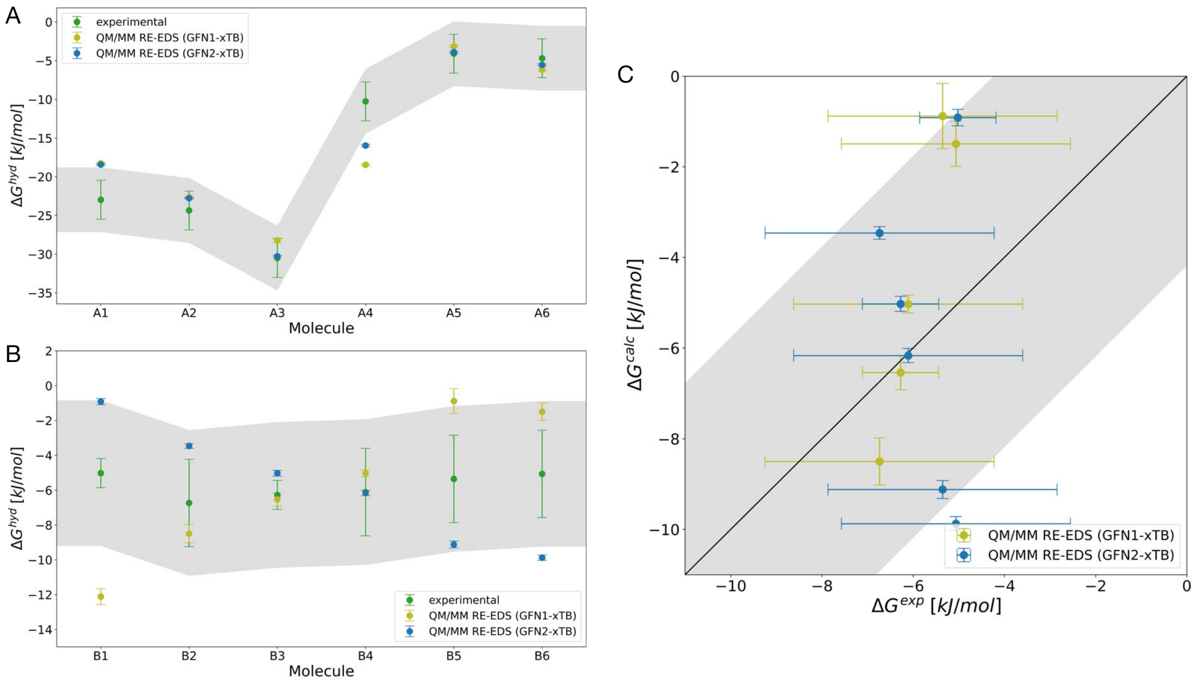

Figure S4: (**A**): Hydration free energy as a function of the molecule identifier for set A for experiment (green), and QM/MM RE-EDS with GFN1-xTB (yellow) and GFN2-xTB (blue). (**B**): Same for set B. (**C**): Correlation with experiment for set B. Error bars represent the standard deviation over repeats and experimental uncertainty for calculated values and experiment, respectively. The shaded grey area depicts the range that falls within ± 4.184 kJ/mol (± 1 kcal/mol) from experimental mean.

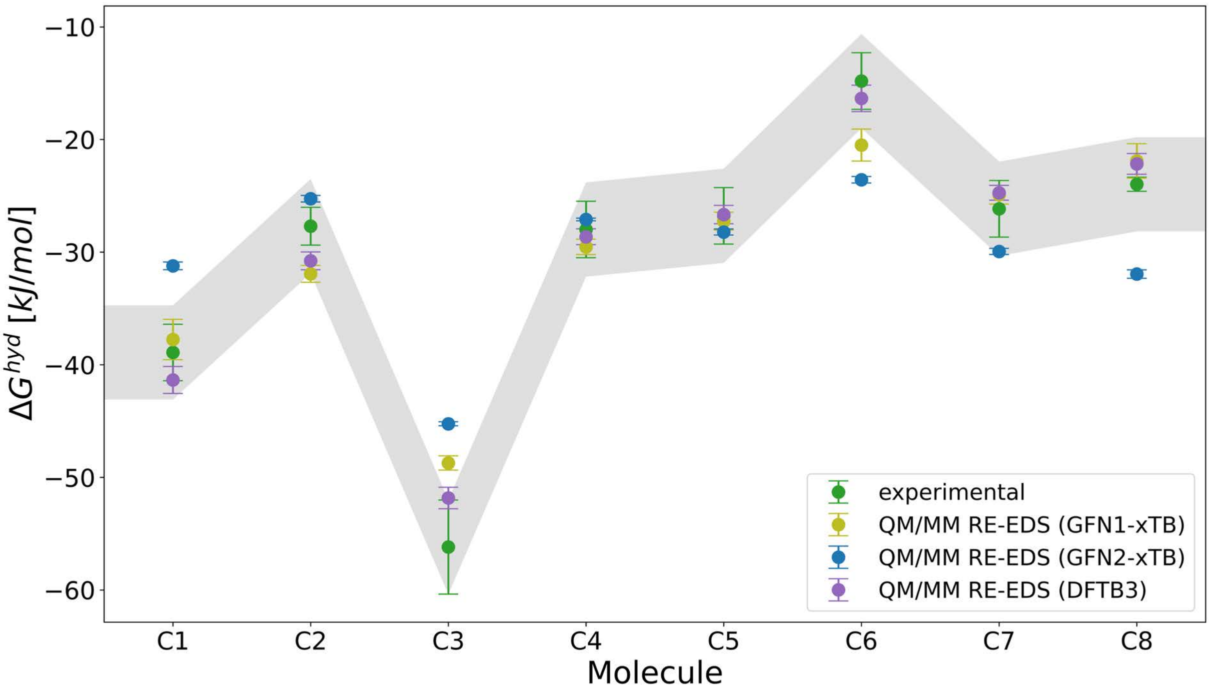

Figure S5: Hydration free energy as a function of the molecule identifier for set C for experiment (green), QM/MM RE-EDS with GFN1-xTB (yellow) and GFN2-xTB (blue), and QM/MM RE-EDS with DFTB3 (purple). Error bars represent the standard deviation over repeats and experimental uncertainty for calculated values and experiment, respectively. The shaded grey area depicts the range that falls within ± 4.184 kJ/mol (± 1 kcal/mol) from experimental mean.

Set	QM Hamiltonian	RMSE [kJ/mol]	R^2	Spearman's ρ	Pearson correlation
A	GFN1-xTB	4.1	0.84	0.94	0.92
	GFN2-xTB	3.1	0.91	1.0	0.96
B	GFN1-xTB	3.8	-33.45	0.09	0.20
	GFN2-xTB	3.3	-25.19	-0.14	-0.26
C	GFN1-xTB	3.8	0.89	0.98	0.97
	GFN2-xTB	6.5	0.68	0.43	0.90
	DFTB3	2.3	0.96	0.98	0.98

Table S2: Comparison of RMSE, R^2, Spearman's ρ and Pearson correlation for all sets.

S3 Compatibility of QM and MM Models

Parameter	TIP3P	SPC/E	OPC3
O charge [e]	-0.8340	-0.8476	-0.8952
H charge [e]	+0.4170	+0.4238	+0.4476
O σ_{LJ} [Å]	3.15061	3.16600	3.17427
O ϵ_{LJ} [kJ/mol]	0.63640	0.65000	0.68369
O-H distance [Å]	0.9572	1.0000	0.9789
H-O-H angle [°]	104.52	109.47	109.47
**Property**	**TIP3P**	**SPC/E**	**OPC3**
Dipole moment [D]	2.35	2.35	2.43
Dielectric constant	82	71	78.4
Self-diffusion [10^{-5} nm^{-2}/ns]	5.19	2.49	2.3
Density maximum [$^{\circ}C$]	-91	-38	-13
Expansion coefficient [$10^{-4}/^{\circ}C$]	9.2	5.14	4.3

Table S3: Comparison of TIP3P [1], SPC/E [2], and OPC3 [3] parameters and selected thermodynamic and dynamic properties.

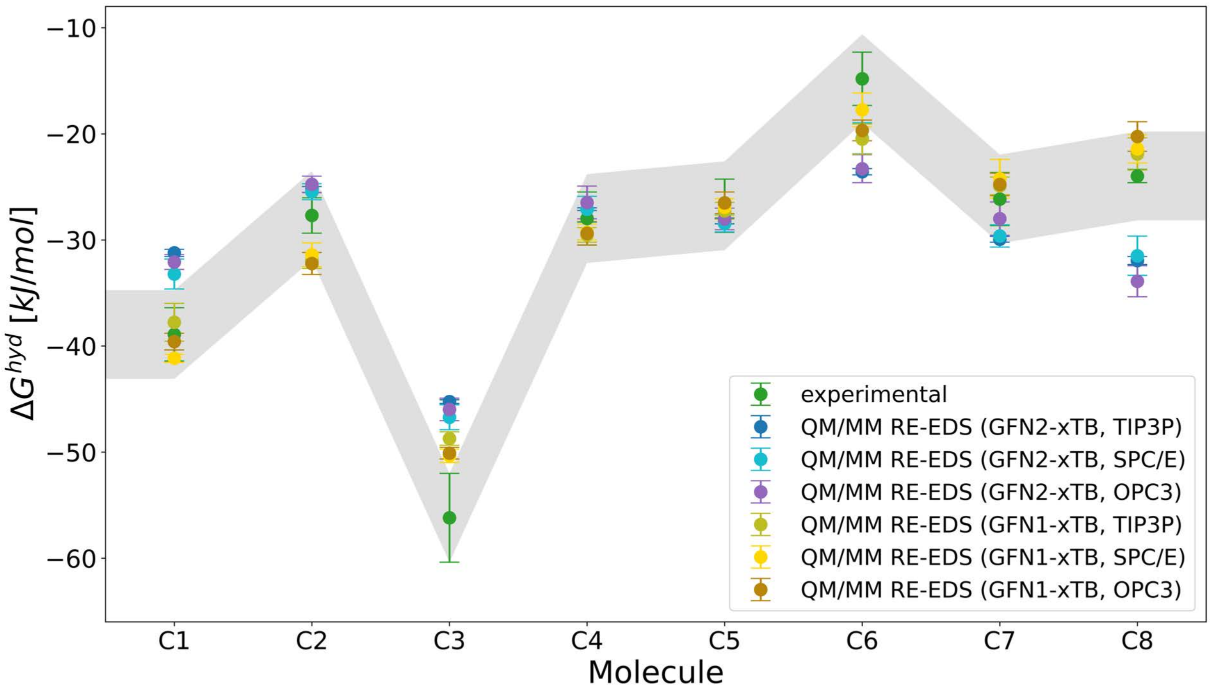

Figure S6: Hydration free energy as a function of the molecule identifier for set C for experiment (green), and QM/MM RE-EDS with GFN1-xTB (yellow colors) and GFN2-xTB (blue colors) and three water models TIP3P, SPC/E, and OPC3. Error bars represent the standard deviation over repeats and experimental uncertainty for calculated values and experiment, respectively. The shaded grey area depicts the range that falls within ± 4.184 kJ/mol (± 1 kcal/mol) from experimental mean.

QM Hamiltonian	Water model	RMSE [kJ/mol]	R^2	Spearman's ρ	Pearson correlation
GFN1-xTB	TIP3P	3.8	0.89	0.98	0.97
	SPC/E	3.0	0.93	0.98	0.97
	OPC3	3.5	0.91	0.98	0.96
GFN2-xTB	TIP3P	6.5	0.68	0.43	0.90
	SPC/E	5.4	0.78	0.55	0.94
	OPC3	6.5	0.68	0.45	0.88

Table S4: Comparison of RMSE, R^2, Spearman's ρ and Pearson correlation for the three water models using set C.

S4 Numerical Values

We provide the numerical values of the experimental and computed hydration free energies for all systems and methods in Tab. S5.

Set A	
**Method**	**Hydration free-energy (molecules 1-6) [kJ/mol]**
experiment	-23.0, -24.4, -30.5, -10.3, -4.1, -4.7
MM MBAR (GAFF, TIP3P)	-24.8, -23.2, -31.8, -11.4, -2.1, -3.6
MM RE-EDS (OpenFF, TIP3P)	-24.8, -23.1, -31.5, -11.6, -2.1, -3.8
QM/MM RE-EDS (GFN1-xTB, TIP3P)	-18.3, -22.6, -28.2, -18.5, -3.1, -6.2
QM/MM RE-EDS (GFN2-xTB, TIP3P)	-18.4, -22.8, -30.3, -16.0, -3.9, -5.5
**Set B**	
**Method**	**Hydration free-energy (molecules 1-6) [kJ/mol]**
experiment	-5.0, -6.7, -6.3, -6.1, -5.4, -5.1
MM MBAR (GAFF, TIP3P)	-8.0, -5.1, -5.3, -5.7, -4.2, -6.3
MM RE-EDS (OpenFF, TIP3P)	-8.2, -5.3, -5.5, -5.8, -3.9, -6.0
QM/MM RE-EDS (GFN1-xTB, TIP3P)	-12.1, -8.5, -6.5, -5.0, -0.9, -1.5
QM/MM RE-EDS (GFN2-xTB, TIP3P)	-0.9, -3.5, -5.0, -6.2, -9.1, -9.9
**Set C**	
**Method**	**Hydration free-energy (molecules 1-8) [kJ/mol]**
experiment	-38.9, -27.7, -56.2, -28.0, -26.8, -14.8, -26.2, -24.0
MM MBAR (GAFF, TIP3P)	-39.0, -28.2, -51.0, -27.0, -25.0, -22.9, -24.7, -24.8
MM RE-EDS (OpenFF, TIP3P)	-41.8, -26.8, -55.5, -25.1, -27.3, -20.5, -23.5, -21.9
QM/MM RE-EDS (GFN1-xTB, TIP3P)	-37.8, -31.9, -48.7, -29.5, -27.3, -20.5, -24.9, -21.9
QM/MM RE-EDS (GFN1-xTB, SPC/E)	-41.2, -31.4, -50.4, -29.3, -26.9, -17.8, -24.2, -21.4
QM/MM RE-EDS (GFN1-xTB, OPC3)	-39.6, -32.2, -50.1, -29.4, -26.5, -19.7, -24.8, -20.3
QM/MM RE-EDS (GFN2-xTB, TIP3P)	-31.2, -25.3, -45.2, -27.1, -28.2, -23.6, -29.9, -32.0
QM/MM RE-EDS (GFN2-xTB, SPC/E)	-33.2, -25.5, -46.7, -27.1, -28.5, -20.4, -29.6, -31.5
QM/MM RE-EDS (GFN2-xTB, OPC3)	-32.1, -24.8, -46.0, -26.5, -28.0, -23.3, -28.0, -33.9
QM/MM RE-EDS (DFTB, TIP3P)	-41.4, -30.8, -51.8, -28.6, -26.7, -16.4, -24.7, -22.2

Table S5: Numerical values for the experimental and mean calculated hydration free-energies over repeats from all simulations.

S5 RE-EDS Related Figures

The most important information to evaluate the convergence of a RE-EDS simulation is discussed in this section. Since two different RE-EDS pipelines were used to match previously obtained results, an example of each is discussed here. First, GFN2-xTB/TIP3P QM/MM RE-EDS for set A, then GFN2-xTB/TIP3P QM/MM RE-EDS for set C. Equivalent behavior was observed for the other simulations.

S5.1 QM/MM RE-EDS with GFN2-xTB + TIP3P QM/MM for Set A

The first step in a RE-EDS pipeline is the optimization of the coordinates for each end-state. This was achieved by performing N EDS simulations with $s = 1.0$ and biasing the system towards a given end-state by adjusting the energy offsets $\mathbf{E}^R$. End-state contributions during this step are shown in Fig. S7.

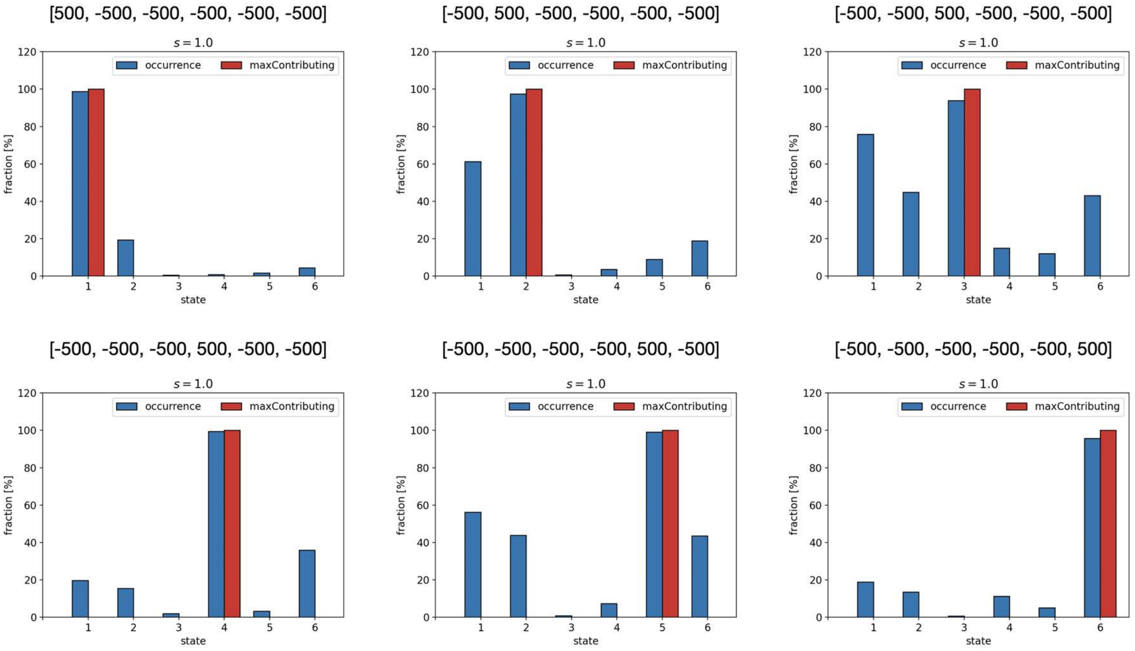

Figure S7: End-state sampling for each of the optimize-state simulations with the applied $\mathbf{E}^R$.

The second step in the pipeline is identifying the lower bound for the s-distribution. This was done by performing 21 EDS simulations with a different smoothing parameter s. The potential-energy timeseries for simulations is shown Fig. S8A. Panel B of Fig. S8 shows the occurrence of each of the six end-states for all simulations. The red line depicts the identified lower bound for s.

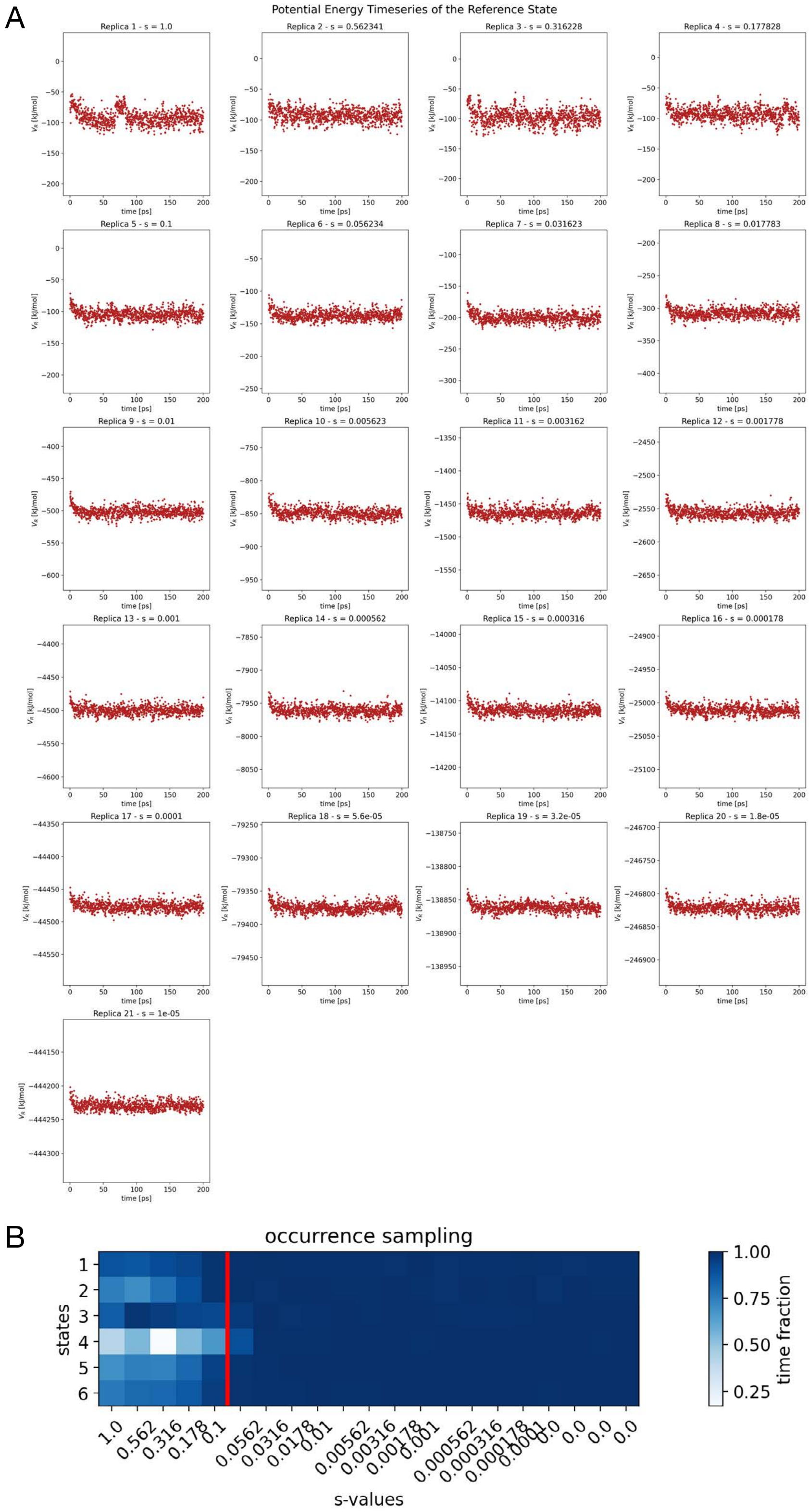

Figure S8: Detection of the lower bound for the s-distribution. (**A**): Time series of the reference-state potential energy. (**B**): Occurrence sampling of all end-states for all 21 simulations. Red line denotes the identified lower bound for s.

In the subsequent energy-offset estimation step, an initial values for the energy offsets $\mathbf{E}^R$ are estimated. 14 s-values were selected from the previous step for the RE-EDS set-up. Since the parameters have not been optimized yet, the replica-exchange frequency and the sampling at $s = 1.0$ are not optimal (Fig. S9A). Panel B of Fig. S9 shows the path each of the trajectories has taken throughout the RE-EDS simulation. Each trajectory has completed a number of round-trips, which means that the s-distribution was reasonable, however, the $\mathbf{E}^R$ need to be optimized (mostly end-state A3 was sampled).

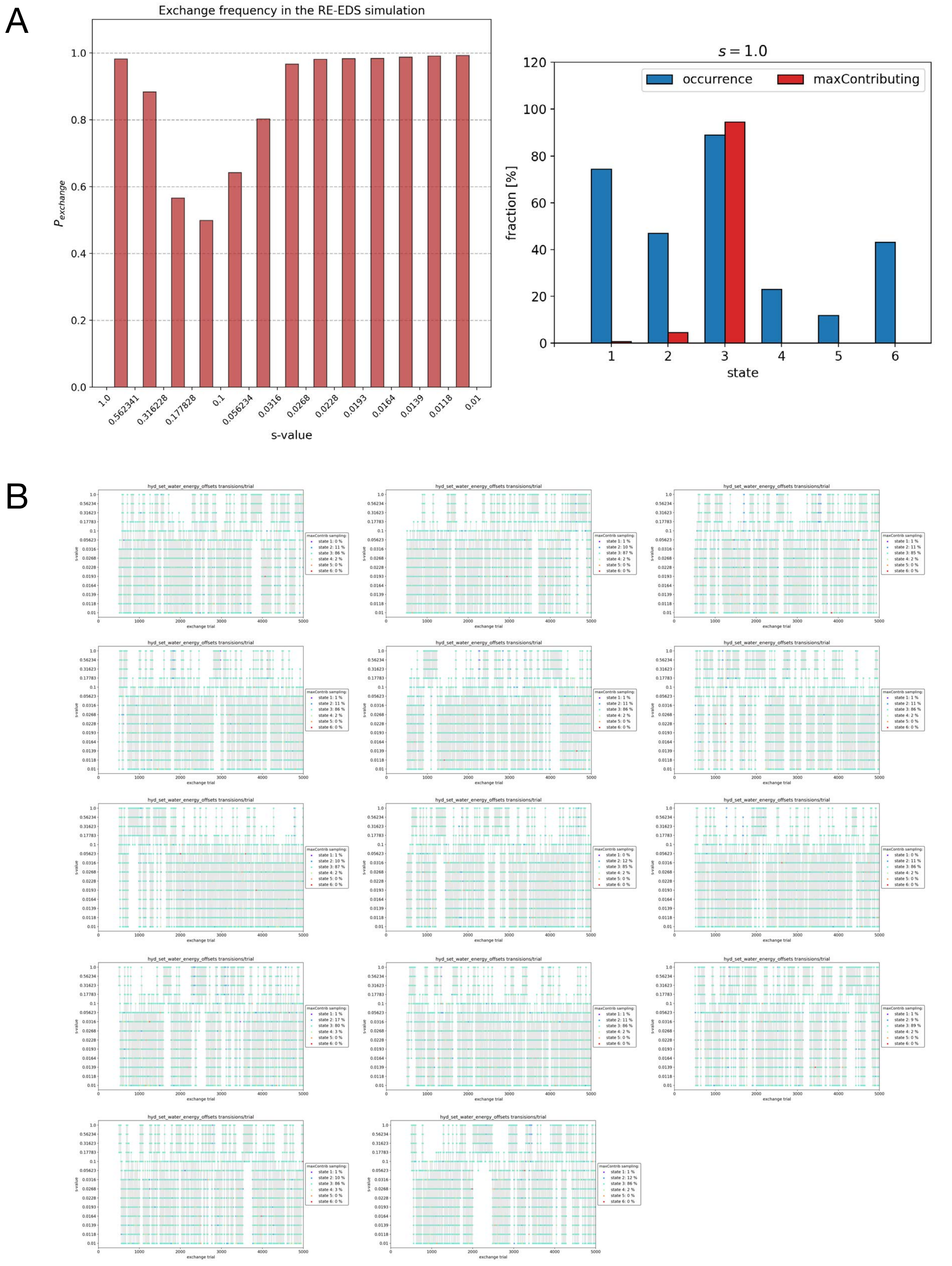

Figure S9: Energy-offset estimation step. (**A**): Replica-exchange acceptance probability (left) and end-state sampling at $s = 1.0$ (right). (**B**): Paths in the replica-exchange space for each of the trajectories.

Next, s-optimization and energy-offset rebalancing steps were performed, each constituting of four iterations. Analysis of the s-optimization and the development of optimal sampling are shown in Fig. S10A. Equivalent plots are shown for the $\mathbf{E}^R$ rebalancing in Fig. S10B.

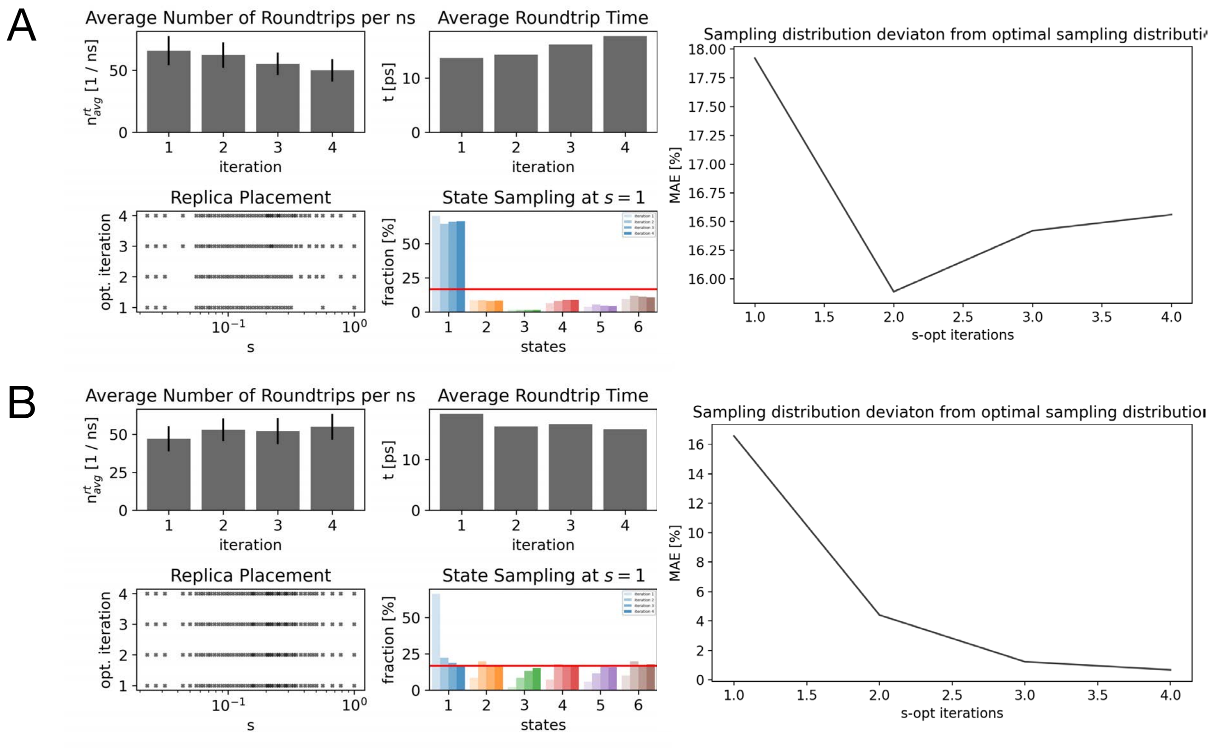

Figure S10: s-optimization and $\mathbf{E}^R$ rebalancing steps. (**A**): Analysis of the s-optimization progress during the four iterations (left) and deviation from optimal end-state sampling per iteration (right). (**B**): Analysis of the $\mathbf{E}^R$ rebalancing progress during the four iterations (left) and deviation from optimal end-state sampling per iteration (right).

After optimal parameters have been selected, ten repeats of the production run were performed with different random seeds for the initial velocities. Here, we show data for one of the repeats. Panel A of Fig. S11 shows the replica-exchange probability, panel B shows the path between replicas taken for one of the trajectories, and panel C shows the convergence of the free-energy differences as a function of the simulation time.

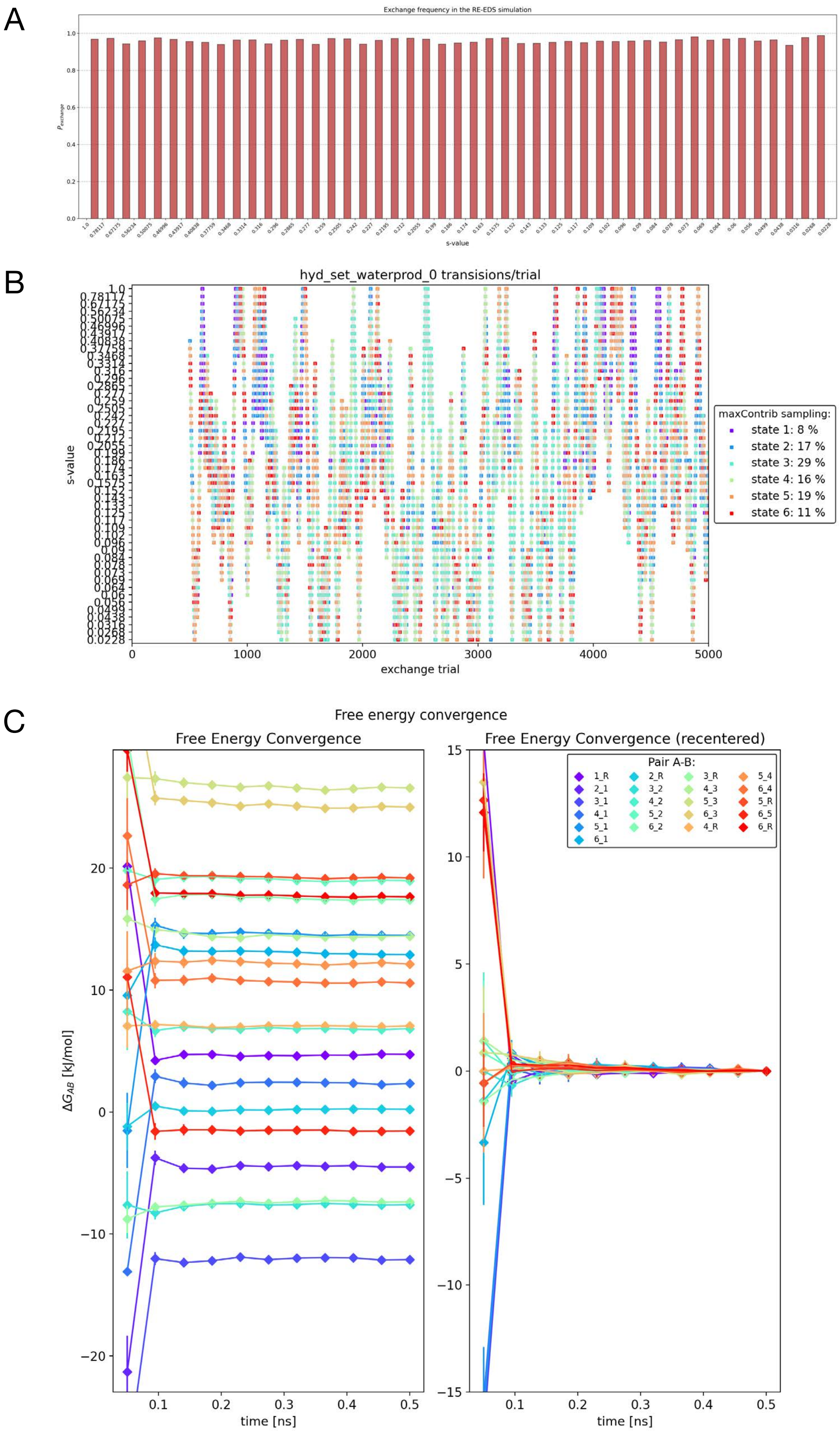

Figure S11: Production run. (**A**): Replica-exchange acceptance probability. (**B**): Path taken and end-states sampled in one of the trajectories. (**C**): Free-energy convergence as a function of simulation time.

S5.2 QM/MM RE-EDS with GFN2-xTB + SPC/E for Set C

Sampling of end-states in each of the EDS simulations performed during the first step of a RE-EDS pipeline is shown in Fig. S12.

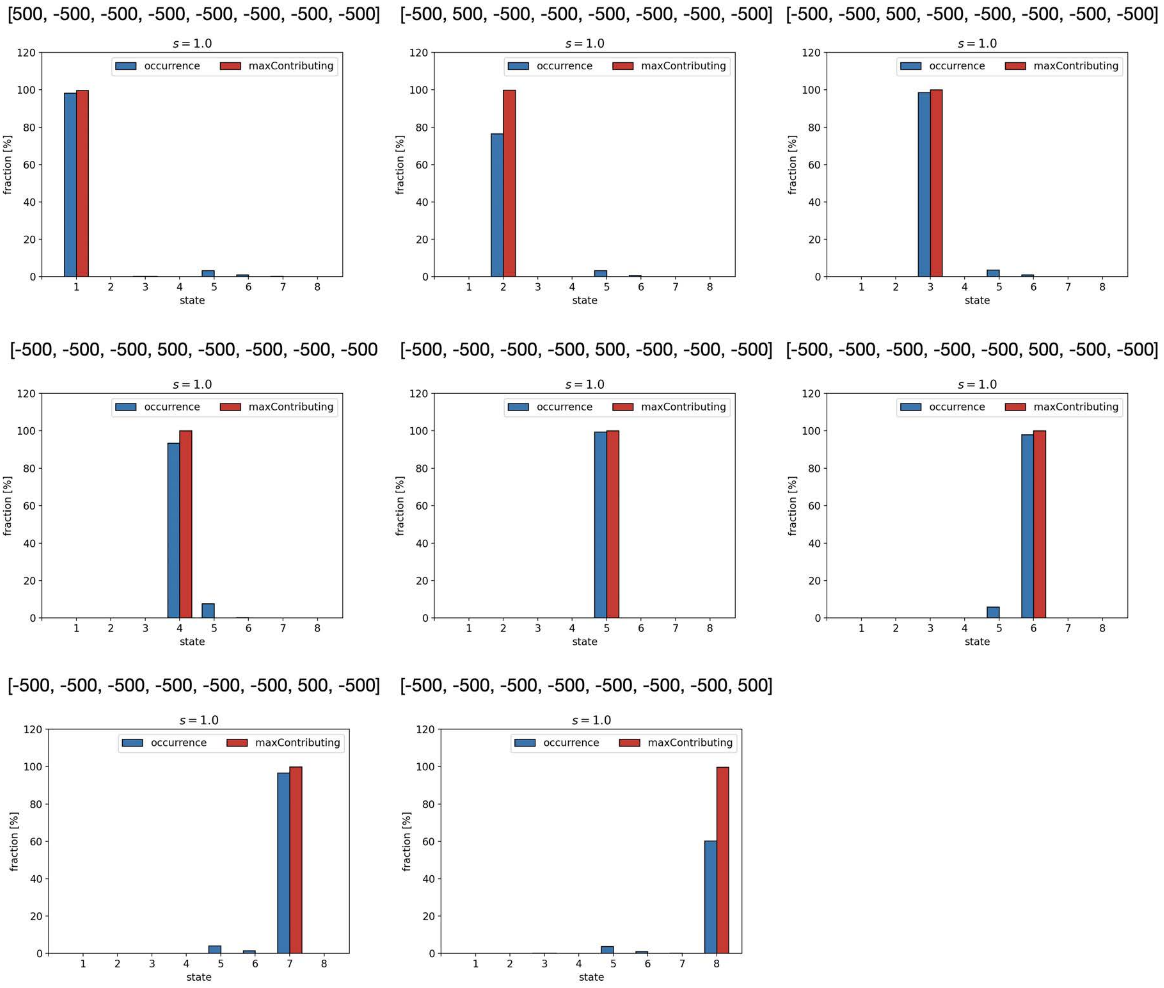

Figure S12: End-state sampling for each of the optimize-state simulations with the applied $\mathbf{E}^R$.

16 s-values were used during the lower-bound detection step and the potential-energy time series are shown in Fig. S13A. Panel B of Fig- S13 shows the occurrence of each of the eight end-states for all simulations. The red line denotes the identified lower bound for s.

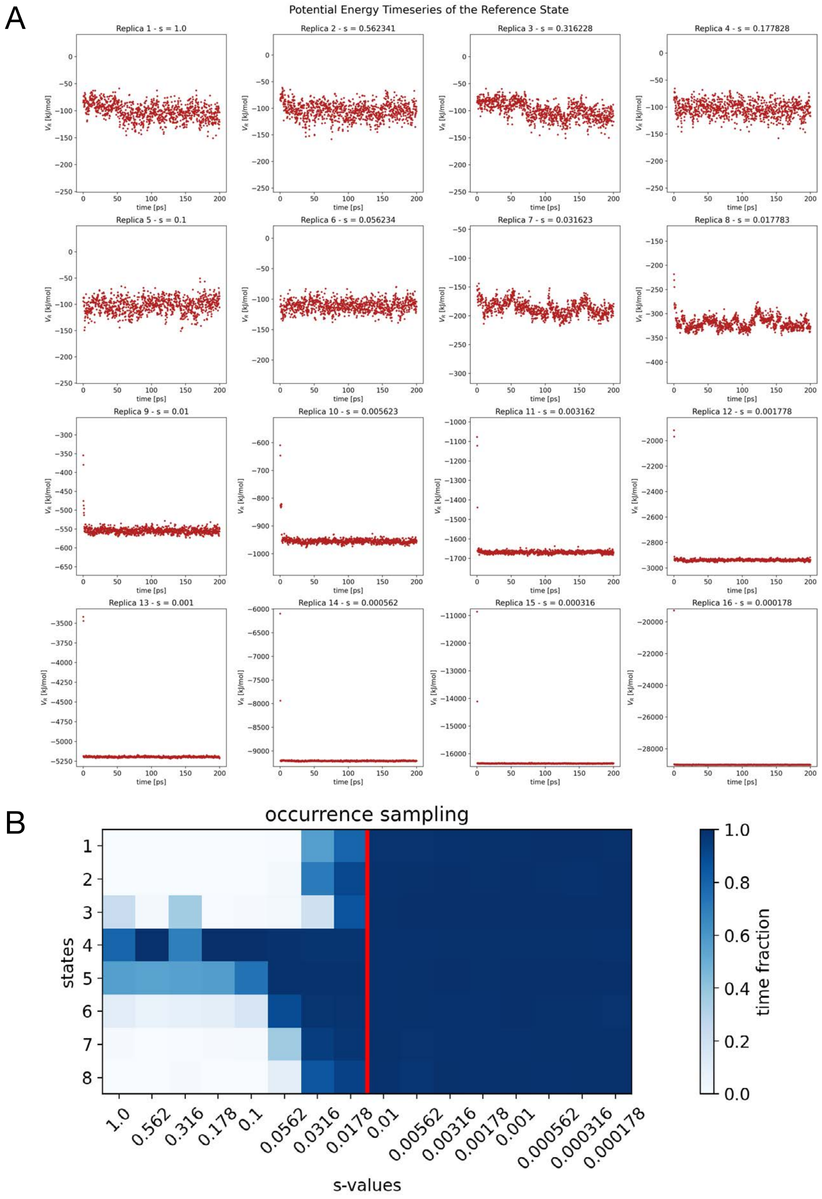

Figure S13: Detection of the lower bound for the s-distribution. (**A**): Time series of the reference-state potential energy. (**B**): Occurrence sampling of all end-states for all simulations. Red line denotes the identified lower bound for s.

17 s-values were used in the initial energy-offset estimation step. The RE-EDS parameters have not been optimized yet and, similarly to the previous example, the replica-exchange frequency at $s = 1.0$ is not yet optimal (Fig. S14A). Panel B of Fig. S14 shows the path each of the trajectories has taken throughout the RE-EDS simulation. Contrary to the example for set A, few round-trips have been completed at this point. Furthermore, not all end-states were sampled at high s-values.

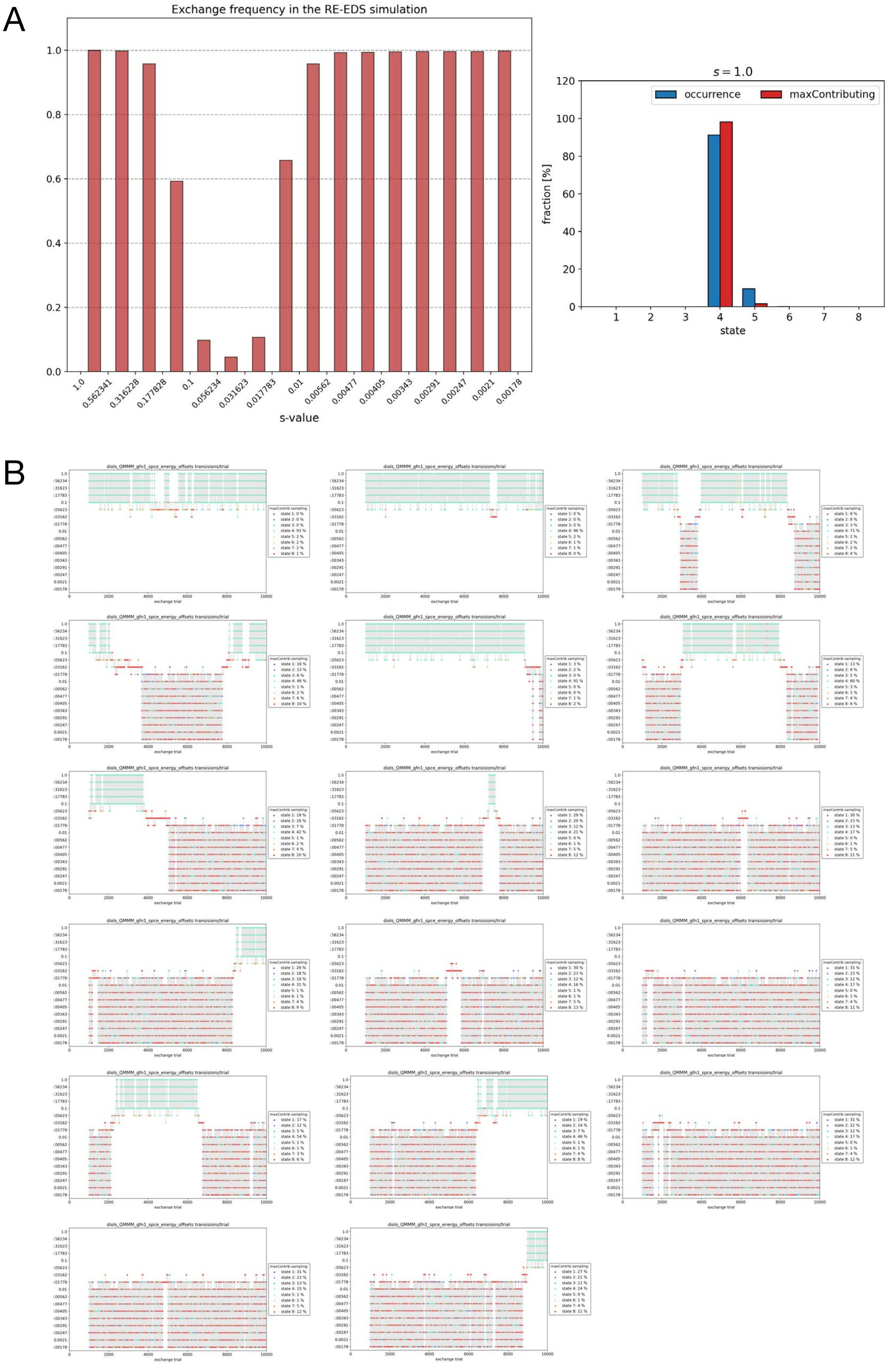

Figure S14: Energy-offset estimation step. (**A**): Replica-exchange acceptance probability (left) and end-state sampling at $s = 1.0$ (right). (**B**): Paths in the replica-exchange space each of the trajectories.

Contrary to the example for set A, a mixed optimization protocol with ten iterations was carried out to optimized the parameters prior to the production runs. Figure S15 shows the analysis of the ten iterations and the development of deviation from optimal sampling.

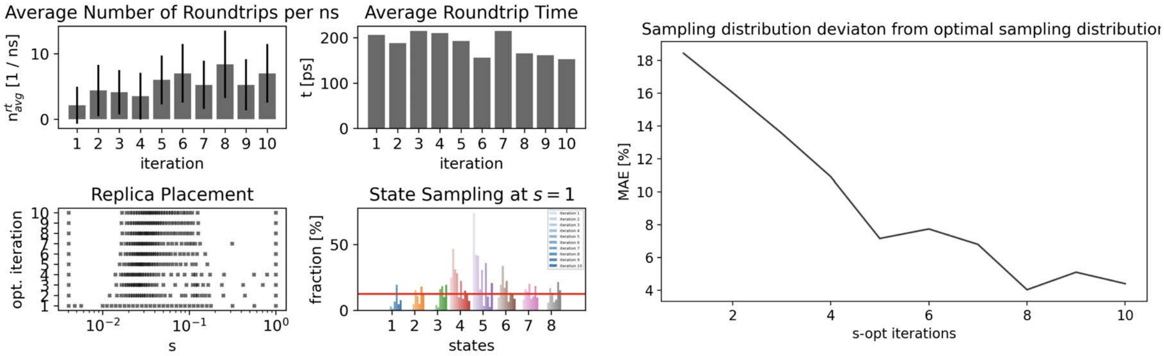

Figure S15: Mixed optimization step. (**A**): Analysis of the s-optimization progress during the ten iterations (left) and deviation from optimal end-state sampling per iteration (right).

After optimal parameters have been selected, ten repeats of the production run were performed with different random seeds for the initial velocities. Here, we show data for one of the repeats. Panel A of Fig. S16 shows the replica-exchange probability, panel B shows the path between replicas taken in one of the trajectories, and panel C shows the end-state sampling in the replica with $s = 1.0$.

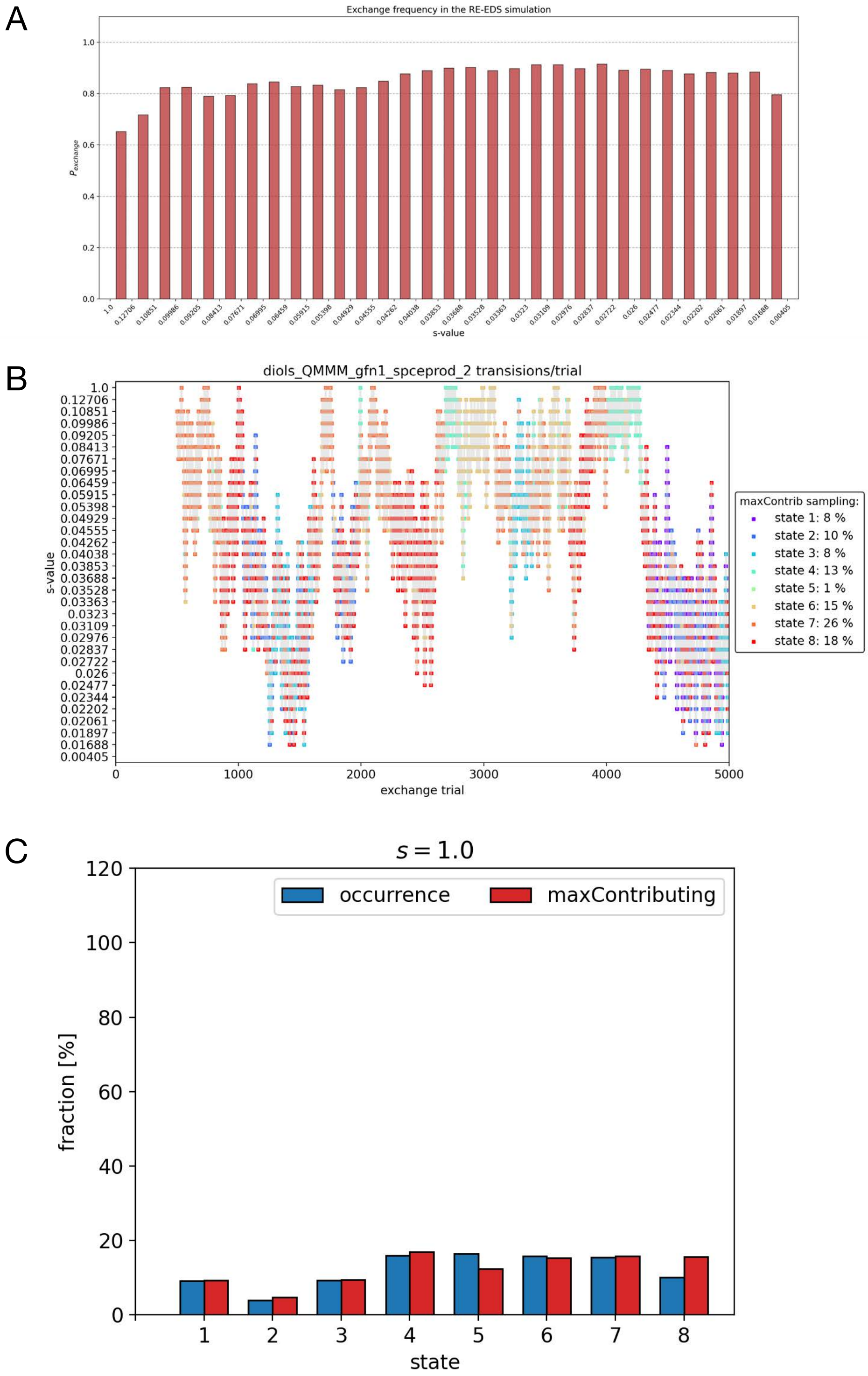

Figure S16: Production run. (**A**): Replica-exchange acceptance probability. (**B**): Path taken and end-states sampled in one of the trajectories. (**C**): End-state sampling at $s = 1.0$.

References

[1] Jorgensen, W. L.; Chandraekhar, J.; Madura, J. D.; Impey, R. W.; Klein, M. L. Comparison of Simple Potential Functions for Simulating Liquid Water. *J. Chem. Phys.* **1983**, *79*, 926–935.

[2] Berendsen, H. J. C.; Grigera, J. R.; Straatsma, T. P. The Missing Term in Effective Pair Potentials. *J. Phys. Chem.* **1987**, *91*, 6269–6271.

[3] Izadi, S.; Onufriev, A. V. Accuracy Limit of Rigid 3-Point Water Models. *J. Chem. Phys.* **2016**, *145*, 074501.
